# Multifunctional Benefits of Prevalent HMOs: Implications for Infant Health

**DOI:** 10.3390/nu13103364

**Published:** 2021-09-25

**Authors:** David R. Hill, Jo May Chow, Rachael H. Buck

**Affiliations:** Abbott Nutrition, 3300 Stelzer Road, Columbus, OH 43219, USA; david.hill1@abbott.com (D.R.H.); jomay.chow@abbott.com (J.M.C.)

**Keywords:** human milk oligosaccharide, breastfeeding, infant formula, pediatric nutrition

## Abstract

Breastfeeding is the best source of nutrition during infancy and is associated with a broad range of health benefits. However, there remains a significant and persistent need for innovations in infant formula that will allow infants to access a wider spectrum of benefits available to breastfed infants. The addition of human milk oligosaccharides (HMOs) to infant formulas represents the most significant innovation in infant nutrition in recent years. Although not a direct source of calories in milk, HMOs serve as potent prebiotics, versatile anti-infective agents, and key support for neurocognitive development. Continuing improvements in food science will facilitate production of a wide range of HMO structures in the years to come. In this review, we evaluate the relationship between HMO structure and functional benefits. We propose that infant formula fortification strategies should aim to recapitulate a broad range of benefits to support digestive health, immunity, and cognitive development associated with HMOs in breastmilk. We conclude that acetylated, fucosylated, and sialylated HMOs likely confer important health benefits through multiple complementary mechanisms of action.

## 1. Introduction

Lactation is a defining feature of all mammals, including humans, and results in the production of milk, a highly specialized food that serves as a complete and sole source of nutrition for breastfed infants. Breastmilk supports optimal growth, digestion, immune defense, cognition, and many other functions necessary for healthy growth and development [1]. Decades of scientific evidence demonstrates that breastfeeding provides the best source of nutrition for infants. For example, breastfed infants have lower rates of respiratory and gastrointestinal infections compared to formula-fed infants [2,3,4]. Breastfeeding is associated with reduced risk of allergy and autoimmune disease in childhood [5] as well as decreased incidence of obesity [6]. Breastfed infants generally exhibit improved cognitive development and may score higher in standardized testing during childhood in comparison to formula-fed infants [7]. These benefits extend well into adulthood, with individuals who were breastfed as infants having generally lower rates of metabolic syndrome and inflammatory disease [5,6]. Thus, the World Health Organization [8] and the American Academy of Pediatrics [9] recommend exclusive breastfeeding for all infants for the first six months of life and have widely advocated for continued breastfeeding of all infants for up to two years.

Despite the consensus that breastfeeding is the best source of nutrition for all infants, breastfeeding is not universally practiced. Social and economic pressures often mean that it is difficult for many women to maintain breastfeeding for a full 12 months [10]. In addition, there are rare but compelling medical circumstances in which breastfeeding may not be advisable or not possible at all, such as the potential for exposure to pharmacologic or chemotherapeutic agents through breastmilk, potential for transmission of infectious disease, or mastectomy [9]. Infant formula is the only suitable, nutritionally complete alternative to breast milk, adapted to provide the nutrients infants need. Thus, there remains a significant and persistent need for innovations in infant formula composition that allow infants to access a wide spectrum of benefits typically associated with breastfeeding.

The addition of human milk oligosaccharides (HMO), structurally and functionally identical to those occurring naturally in human breast milk, represents the most significant innovation in infant formula technology of the past decade. Only very recently have advances in the large-scale production of HMOs made fortification of infant formula feasible [11,12]. There exists a growing body of scientific literature on the safety and benefits of HMO fortification [13,14,15,16], notably 2′-fucosyllactose (2′-FL) [17,18,19,20,21], the first HMO to be widely added to commercial infant formula, which is now available from several manufacturers. Researchers have identified as many as 150 unique HMOs occurring naturally in human milk at widely varying concentrations and prevalence [22,23]. Continuing innovation in the bulk synthesis of HMOs will likely enable fortification of infant formula with more complex mixtures of HMO structures in the near future, expanding the range of benefits for infants and more closely replicating the function of breastmilk.

This review will characterize the structure of HMOs in human breastmilk and will propose a set of core HMOs that recapitulate a wide range of the HMO functions that are provided naturally in breastmilk. We summarize the scientific literature demonstrating that these HMOs serve critical functions in supporting the developing immune system, promoting digestive health and development of the gut microbiome, and supporting cognitive function.

## 2. HMO Structure and Composition

HMOs are defined as a carbohydrate of one lactose disaccharide covalently bound to one or more additional monosaccharides or disaccharides. Lactose serves as the essential base upon which a wide range of biochemical modifications are affixed. This lactose subunit is modified by the terminal monosaccharides fucose or sialic acid (i.e., N-acetylneuraminic acid), or it may be extended by the addition of lacto-N-biose (Galβ1-3GlcNAc) and/or N-acetyllactosamine (Galβ1-4GlcNAc) disaccharides. The addition of terminal monosaccharides to either lactose or a complex HMO of lactose plus lacto-N-biose and/or N-acetyllactosamine terminates the carbohydrate chain and prevents any further glycans from being added to that branch of the structure. Because the addition of one or more sialic acid residues introduces a negatively charged carboxyl group, the pool of HMOs with terminal sialic acid residues are commonly referred to as acidic or **sialylated** HMOs. By contrast, neutral HMOs terminate in fucose (**fucosylated** HMO) or N-acetylglucosamine (GlcNAc i.e., **acetylated** HMO).

The lactose core may or may not also be elongated by one or more units of the disaccharides lacto-N-biose (Galβ1-3GlcNAc) and/or N-acetyllactosamine (Galβ1-4GlcNAc) in either a linear or branching pattern. This enables the addition of multiple fucose or sialic acid residues at the terminal positions or at the branching C6 position of galactose, as seen in disialyllacto-N-tetraose (DSLNT) [24]. Notably, while the two elongating disaccharide structures differ only in the nature of the linkages between galactose and N-acetylglucosamine (C1 to C3 vs. C1 to C4), this linkage may determine whether the HMO is subject to degradation by lactase expressed at the brush border of the proximal small intestine [25]. The β1-3 linkage is not a substrate for lactase or any other enzyme produced by the host tissues of the infant gastrointestinal tract and is therefore presented intact to the intestinal microbiota; the β1-4 linkage is a substrate for lactase and is primarily cleaved by digestive enzymes in the proximal small intestine. This may have significant consequences for the presentation of HMO structures in the gut [25,26]. For example, while lacto-N-tetraose (LNT) and lacto-N-neotetraose (LNnT) are isomers of the same four monosaccharides, the presence of a β1-4 linkage in LNnT may make the structure susceptible to cleavage by endogenous or microbial lactases in the proximal small intestine [25]. Conversely, LNT passes intact to the distal GI tract, where it is available as a substrate for microbial fermentation or as a ligand for immune modulation [27]. Thus, from a small pool of components (lactose, fucose, sialic acid, lacto-N-biose, and N-acetyllactosamine), we derive at least 150 unique HMO structures found in nature [22]. There are likely additional rare or low abundance variants that occur naturally but have not yet been characterized.

HMOs are distinct from all other dietary carbohydrates in that their only natural source is breastmilk. Bovine milk oligosaccharides (BMOs) share some structural similarities with HMOs but occur at far lower concentrations [28,29,30]. Human evolutionary history has exerted strong selective pressure that shaped both the maternal production of HMOs and the utilization of HMOs by the nursing infant in favor of enhanced reproduction and survival [31,32]. HMOs are naturally synthesized by the mammary epithelium during lactation and are secreted in milk at a total concentration of roughly 5–15 g/L [25,33,34]. As other reviewers have commonly noted, this makes HMOs the third most abundant solid component of human milk following free lactose and lipids. Synthesis of indigestible oligosaccharides is energetically costly for the nursing mother and diverts the calories contained in lactose into an alternate form that cannot be directly metabolized [35]. This implies that HMOs have non-nutritional functions that are highly adaptive and beneficial to the developing infant. Indeed, many such functions have now been identified and are reviewed below in the section “Essential Functions of HMOs”. 

While natural selection has strongly favored the presence of the robust and diverse pool of HMOs found in all human milk, the specific composition of human milk and HMOs varies widely between individuals and across global populations [22,36,37,38,39]. Other reviewers have assembled exemplary summaries of the various studies measuring concentrations of HMOs in human milk [22]. At least a portion of this variation is determined by genetic factors. Discrete genetically encoded traits are known to determine the frequency of terminal fucose residues in HMOs and mucosal glycoproteins. Two genes encoding fucosyltransferase enzymes, FUT2 and FUT3, transfer fucose to carbohydrate chains at different linkages: FUT2 (i.e., the Secretor gene) adds fucose to galactose with an α1-2 linkage [40], and FUT3 (i.e., the Lewis gene) adds fucose to glucose or N-acetylglucosamine in α1-3 or α1-4 linkages [41]. Secretor and Lewis polymorphisms occur at variable frequency across different populations and have a complex evolutionary history resulting from competing selective pressures [42]. FUT2 polymorphisms are associated with protection from some infectious diseases as well as increased susceptibility to others [43,44,45,46,47,48,49]. There have also been notable reports in which Secretor status was shown to have no significant impact on clinical outcomes [50].

Beyond the genetics that determine the potential for fucosylation of HMOs, other aspects shaping differences in HMO structure and composition in milk are less clear. There is some evidence to suggest that maternal diet and exercise may play a role in breastmilk HMO composition [25,51]. Studies that draw a direct relationship between maternal environment or lifestyle, HMO composition, and pediatric outcomes are inherently difficult to conduct. There has been recent progress in this area [52,53], including substantial associations between HMO composition and maternal diet [53]. Several researchers have speculated that HMO composition is uniquely tailored by mothers to meet the needs of their individual babies over time, although the specific genetic and enzymatic mechanisms that would be required to regulate the synthesis of HMOs in the mammary gland to this level of specificity have yet to be described. Mothers and their children have at least a 25% chance of sharing the same Secretor and Lewis phenotypes [54]; beyond this, the means by which breastmilk HMO composition is matched to the needs of the infant requires further investigation and the clear distinction of such interactions from the influence of a shared maternal–infant environment. Indeed, we propose that it is not yet clear that all the factors shaping the HMO pool composition are specifically regulated. There may be sufficient tolerance and redundancy that has evolved within the mother–infant feeding dynamic such that as long as a diverse pool of HMO structures are present in the diet, a stable microbial and immunological phenotype is likely to develop in early life. This would be consistent with the consistently positive outcomes associated with milk banking, in which the donor and recipient have no shared home environment, close genetic relationship, or personal contact [55,56].

### Core HMOs

**Figure 1:**
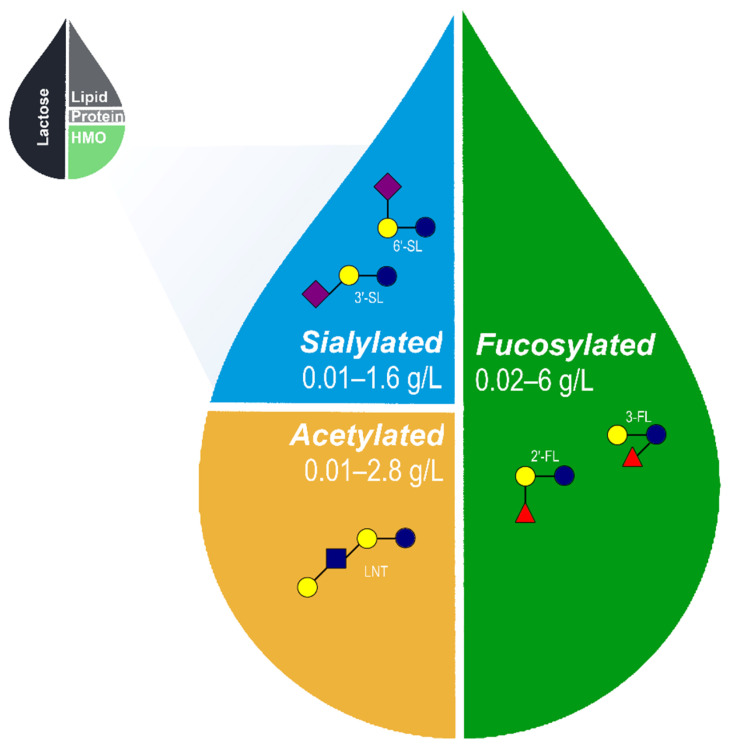
Approximate relative proportions of macronutrients in human breastmilk and range of acetylated, sialylated, and fucosylated HMOs naturally occurring in breastmilk [22,23,57,58,59,60,61,62]. The proposed set of 5 core HMOs used in the fortification of infant formula likely recapitulates a wide range of benefits associated with HMOs in human breastmilk. HMO, human milk oligosaccharides; 2′-FL, 2′-fucosyllactose; 3-FL, 3-fucosyllactose; 3′-SL, 3′-sialyllactose; 6′-SL, 6′-sialyllactose; and LNT, lacto-N-tetraose.

What then constitutes the necessary subset of the HMO pool required to perform the full range of functions attributed to HMOs in breastmilk (Figure 1)? Given the current incomplete state of our understanding of HMO structure and function, defining such a set of core HMOs will certainly remain an active and dynamic area of investigation for some time to come. Yet, this effort is both necessary and justified within the broader goals of pediatric nutrition in that it provides a target for future research and innovation and may be used as a basis for improved infant formula fortification strategies. We define the term Core HMOs as follows: among all possible HMOs found in nature, a subset of specific structures composed of acetylated, sialylated, and fucosylated HMOs that provide the range of functional benefits attributed to the HMOs found in breastmilk. Therefore, any proposed set of Core HMOs must be necessarily guided by an examination of the functions of HMOs. In the next section, we will examine the essential functions of HMOs and the specific HMO structures that perform those functions.

## 3. Essential Functions of HMOs

**Figure 2:**
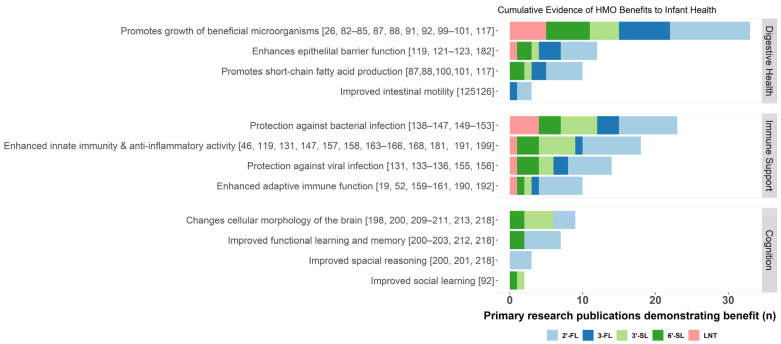
Summary of known benefits of acetylated (red), fucosylated (blue), and sialylated (green) HMOs. The cumulative evidence for each potential benefit is approximated by the number of independent primary research publications demonstrating evidence of an association between a given HMO and health benefit, indicated here by a stacked bar chart colored by HMO structure. HMO, human milk oligosaccharides; 2′-FL, 2′-fucosyllactose; 3-FL, 3-fucosyllactose; 3′-SL, 3′-sialyllactose; 6′-SL, 6′-sialyllactose; and LNT, lacto-N-tetraose.

HMOs are energetically costly for the mother to produce and secrete in breastmilk yet provide few direct calories for her infant [31,32]. At the heart of HMO research is the mystery of why a system would evolve that seemingly diverts calorie-rich lactose and other carbohydrates to produce indigestible HMOs. Milk oligosaccharides are highly conserved across all mammals [30,31,32,63], suggesting that the presence of these structures in milk confers selective advantages. A growing body of evidence has outlined functions of HMOs in promoting gut health in the protection from pathogens and immune disease and in cognitive development (Figure 2). Collectively, HMOs ensure robust growth and development in these systems through multiple complementary structures and diverse mechanisms of action.

### 3.1. Digestive Health

Ingested HMOs exert multiple direct and indirect benefits as they pass through the gastrointestinal tract. Perhaps most important is their influence on the assembly and function of the community of microorganisms that inhabits the gastrointestinal tract, the gut microbiome. HMOs have also been shown to support robust intestinal barrier function and to regulate gut motility. Collectively, these mechanisms of action ensure complete nutrition and immune protection that enables healthy growth and development in infancy.

#### 3.1.1. Prebiotic Functions of HMOs 

The human microbiome encompasses a vast diversity of microorganisms that exist within and on our bodies and that integrate in essential ways with our physiology. Research over the past decade has revealed that the gut microbiota plays a key role in human health and disease [64,65]. Trillions of microorganisms within the digestive tract process ingested food for maximum nutritional benefit. In addition, intestinal bacteria perform essential functions in the maturation and education of the immune system, protection against pathogens, regulation of intestinal endocrine functions and neurologic signaling, and provision of vast metabolic capabilities [64]. Infancy represents a critical window for the establishment of the host–microbe symbiosis within the gastrointestinal tract, with mode of delivery, nutrition, and environmental exposure to bacteria collectively shaping the developing microbial community in the newborn [66]. Disruptions in the formation of a stable intestinal microbiota promote inflammation in premature infants [67,68,69] and may contribute to the development of allergy [70,71], asthma [72], obesity and diabetes [73,74], and inflammatory bowel disease later in life [75,76]. Thus, it is critical to provide all infants with an environment that supports healthy microbiota development.

Nutrition remains perhaps the most significant modifiable factor in promoting the formation of a healthy microbiota [77,78]. It has long been understood that breastfed infants harbor microbial communities that are distinct from their formula fed peers, and this difference in microbial colonization may underlie some of the differences in health outcomes associated with mode of feeding in infancy [79,80]. In recent years, HMOs have been revealed as unique, multifunctional candidate prebiotic carbohydrates, or “substrate[s] selectively utilized by host microorganisms conferring a health benefit” [81]. Numerous preclinical studies and emerging clinical data have demonstrated the highly selective utilization of HMOs by the infant gut microbiota and the resulting competitive advantage to organisms that can metabolize these compounds. For example, beneficial microbes *Bifidobacterium infantis* and *B. bifidum* express specialized enzymes that allow for the rapid consumption of fucosylated, acetylated, and sialylated HMOs and which are not commonly found in other gut-associated bifidobacteria [26,82,83,84]. Other *Bifidobacterium* species, namely *B. breve,* have specialized in the metabolism of the acetylated HMOs LNT and LNnT [85]. *Bacteroides* species, including *B. thetaiotamicron*, which are characteristic of the healthy mature gastrointestinal tract and provide robust metabolic and immune support, are also proficient HMO degraders [86,87,88]. By contrast, other gut microbes and opportunistic pathogens, such as *E. coli*, *C. perfringens*, *Staphylococcus* spp., and *Enterobacter* spp., do not utilize HMOs and may be inhibited by the fermentation products of HMO-degrading bifidobacteria [87,88]. In the highly competitive microbial environment of the developing gastrointestinal tract, this advantage in HMO utilization dramatically impacts the survival and persistence of beneficial *Bifidobacterium* species and reduces the burden of potentially detrimental or infectious environmental bacteria [89]. The presence of fucosylated HMOs in breastmilk has been directly associated with changes in microbial composition [90]. This relationship between HMO consumption and gut microbial community composition may extend to formula-fed infants as well. Recently, infants fed formula containing 2′-FL and LNnT were shown to be more likely to harbor a bifidobacteria-rich microbial community relative to infants fed standard formula [91]. Furthermore, the study found that infants harboring high levels of bifidobacteria at three months of age were less likely to be prescribed with antibiotics during the first year of life [91]. Likewise, supplementation of laboratory mouse diets with either 6′-SL or 3′-SL for up to 20 weeks led to substantial alterations in the major phlya in samples of colonic contents [92]. Finally, the presence of *Bifidobacterium* and *Bacteroides* spp. with the ability to use specific glycan structures that are shared between intestinal mucin and milk provides added benefits for the HMO-consuming infant. This capability, which belongs to certain *Bacteroides* spp., may result in a more stable microbial community with a wider spectrum of enzymatic capabilities that aid in the transition to a solid food diet after weaning [93]. Taken together, dietary HMOs clearly provide direct support for the expansion of health-associated bacteria in the developing gastrointestinal tract and likely confer associated health benefits.

The presence of HMOs also supports cross-feeding, a phenomenon in which metabolic byproducts produced by one bacterium serve as a nutrient source for another type of bacterium present in the ecosystem [94]. Utilization of HMOs by species such as *B. bifidum* supports cross-feeding among bifidobacteria and between bifidobacteria and other genera. *B. bifidum* externally degrades diverse HMO structures into a variety of metabolites [95] that support other bifidobacteria species, thus promoting diversity and dominance of this genus during early life [96]. Utilization of HMOs by *B. bifidum* also supports cross-feeding between bifidobacteria and butyrate-producing bacteria that convert the co-substrates, acetate and lactate, to butyrate, a short-chain fatty acid (SCFA) that plays a critical role in host health [97,98]. In vitro experiments examining infant fecal microbiota cultured in the presence of HMOs demonstrate decreasing concentrations of lactate coinciding with the accumulation of butyrate have been interpreted as evidence of cross-feeding between HMO-consuming bifidobacteria and other butyrate-producing bacterial species [99,100,101]. Lactobacilli, which commonly inhabit the infant gut [102], participate in HMO degradation by scavenging of mono- and disaccharides generated by other bacterial genera rather than degrading intact oligosaccharides [103]. In general, *Lactobacillus* species have limited ability to degrade intact HMOs [84,86,88], but some strains, such as *L. acidophilus* NCFM, have been shown to grow on mixtures of HMOs, presumably on acetylated structures, such as LNnT [84]. Bacterial growth experiments also revealed that the ability to utilize N-acetylglucosamine was widespread among probiotic- and human-associated *Lactobacillus* strains, while the ability to grow on fucose [84,104] and sialic acid is far less common [103]. These findings point to both the direct prebiotic activity of HMOs in supporting the growth of bifidobacteria and the added benefits of the cross-feeding mutualist relationships that this nutritional support fosters.

#### 3.1.2. Generation of Beneficial Microbial Metabolites

The metabolic byproducts of HMO fermentation and microbial cross-feeding also have direct benefits for the infant intestine. SCFAs, such as acetate, proprionate, and butyrate, exert multiple beneficial physiological effects in the host: acting as anti-inflammatory agents, supporting the barrier function of intestinal epithelial cells, serving as modulators of chemotaxis and adhesion of immune cells, promoting GI motility through direct inhibition of G-coupled-receptors, inhibiting histone deacetylases, and serving as energy substrates for intestinal epithelial cells [97,105]. SCFAs produced by *Bifidobacterium* and cross-fed microbial communities also act as general growth suppressants for a variety of gut pathogens [87,106]. These same SCFAs may slow bacterial translocation from the intestine, limiting the scope of infection through enhanced epithelial barrier function [107]. Other metabolites of *B. bifidum* and *B. crudilactis* produced from fermented 3′-SL suppress expression of virulence factors found in *E. coli* and *Salmonella* [108]. The presence of *Bifidobacterium* is associated with broad changes in gene expression within the neonatal intestine [109]. Preclinical experiments have repeatedly demonstrated that *B. infantis* and its fermentation products act to reduce intestinal permeability and support intestinal barrier function [110,111,112,113]. These effects may be at least partially explained by the ability of *B. infantis* to secrete indole-3-lactic acid (ILA), a specialized tryptophan metabolite with specific anti-inflammatory effects only in immature intestinal enterocytes [114]. *B. infantis* grown in the presence of HMOs appear to be particularly effective in promoting epithelial barrier integrity and anti-inflammatory immune signaling [113,115].

Changes in the microbiota fueled by the presence of HMOs may contribute to long-term changes in the immune environment that protect against autoimmune or metabolic disease. For example, provision of HMOs prevented the development of severe pancreatic insulinitis in a mouse model of type 1 diabetes by promoting beneficial changes in the composition of the intestinal microbiota that resulted in increased SCFA production and the expansion of tolerogenic T regulatory cells and dendritic cells [116]. Likewise, 2′-FL feeding significantly decreased the severity of colitis in *Il10*-deficient mice in part through the expansion of commensal microbes and increased production of SCFA [117]. Finally, 2′-FL feeding was shown to reduce weight gain in mice fed a high-fat diet and was associated with changes in microbiome composition, immune signaling, adipogenesis, and the production of satiety signals in the gut [118]. It is therefore evident that HMOs promote the development of a *Bifidobacterium*-enriched intestinal microbiome that provides protection from infection, enhances epithelial barrier function, and produces beneficial immunomodulatory metabolites. These changes in the developing intestinal environment may contribute to long-term protection from autoimmune, metabolic, or inflammatory disease.

#### 3.1.3. Intestinal Barrier Protection

Recently, direct benefits of HMOs on gut function have been described which complement the broad indirect benefits resulting from microbial fermentation of HMOs. 3-FL stimulated production of mucin and antimicrobial peptides in goblet cells, and 2′-FL may have a similar effect on goblet cell function when inflammatory stressors are also present [119]. Animal models have reported similar effects for pooled HMOs [120]. Both 2′-FL and 3-FL appear to also stimulate maturation of the glycocalyx, a thin layer of glycoproteins on the epithelial cell surface that underlies the intestinal mucus barrier [121]. 2′-FL and 6′-SL appear to have divergent but potentially complementary effects on cell proliferation in culture models, suggesting that HMOs may confer the most effective benefits when multiple structures are delivered in concert [122]. Natividad et al. tested this hypothesis by examining the effects of a variety of fucosylated, sialylated, and acetylated HMO structures both individually and in combinations of up to six structures for their ability to modify epithelial barrier function. They found that a combination of fucosylated, sialylated, and acetylated HMOs at proportions comparable to levels in human milk enhanced intestinal barrier integrity under inflammatory conditions. Subsequent experiments with individual HMOs revealed that much of the effect could be attributed to 2′-FL alone [123]. These effects on mucus production and goblet cell differentiation may also reflect altered enteroendocrine and enteric nervous system function, which is integral to mucus secretion [124]. Ex vivo experiments using rodent tissues indicate that fucosylated HMOs modulate intestinal motility. Intestinal segments submerged in buffer containing either 2′-FL or 3-FL reduced the frequency, velocity and amplitude of contractions, while lactose, galactooligosaccharide (GOS), LNnT, 3′-SL, and 6′-SL had little if any effect. Fucose alone also decreased contractility but to a much lesser degree than the fucosylated HMOs [125]. In a follow-up study using the same model but with intestinal segments harvested from mice subjected to acute restraint stress, Farhin et al. demonstrated that stress reduced propulsive motility in the small intestine and that it could be reversed by perfusing the segments with 2′-FL. The stress-induced increase in colonic motility was similarly mitigated by treating with 2′-FL [126].

HMOs confer substantial benefits to digestive health through both indirect support to the microbiome and direct action on the intestinal mucosa itself. The full implications of these benefits for developing infants remains to be determined, but it is clear that supporting microbiome health and digestion is an essential component to establishing complete nutrition and immune homeostasis. Multiple fucosylated, acetylated, and sialylated HMOs act in concert to deliver these benefits, and fortification of infant formula with diverse HMOs representing each of the major categories may be the most effective strategy to deliver digestive health support comparable to breastfeeding.

### 3.2. Immune Support

HMOs act to support immunity and immune development through mechanisms of action that directly target potential pathogens as well as mechanisms that modulate the behavior of immune cells or promote beneficial immune signaling [1,25,33]. Collectively, these multiple actions seem to underlie the beneficial effects of HMOs in animal models of necrotizing enterocolitis, allergy, and autoimmunity. Future studies may expand on the potential contributions of HMOs to other aspects of immune development.

#### 3.2.1. Direct Anti-Infective Activity

Viral pathogens are widespread in human populations [127] and particularly among infants [128,129,130]. All viruses must obtain entry to host cells in order to replicate and spread to new hosts, often causing disease in the process. This requires the virus, which circulates passively, to bind to structures on the surface of appropriate host cells. Disruption of this process is key to the passive immunity conferred to breastfeeding infants [3]. Viral binding targets are frequently composed of oligosaccharide moieties, which are widespread within the intestinal mucosa and serve as a buffer to the external environment. HMOs share extensive structural similarities with the glycans that compose the epithelial mucus layer [33]. Soluble HMOs occupy binding sites present on the surface of viral particles, substantially reducing the cumulative avidity of a viral particle towards its target host cell [1]. Multiple HMOs exhibit direct antiviral activity (Table 1). In epithelial cell cultures, 2′-FL and 3′-SL significantly inhibit the replication of respiratory syncytial virus [131], a major viral pathogen affecting the lower respiratory tract in infants [132]. Likewise, 6′-SL was shown to inhibit the replication of influenza A strain H1N1 in cultured respiratory epithelial cells in a dose-dependent manner [131]. HMOs also exhibit inhibitory activity against gastrointestinal viruses [133]. Sialylated HMOs including 3′-SL and 6′-SL have been shown to inhibit rotavirus infection in vitro and in animal models [134]. In groundbreaking work, Weichert et al. [135] demonstrated that both 2′-FL and 3-FL inhibit the binding of norovirus surface proteins to target receptors and elucidated the functional basis of this observation: 2′-FL and 3-FL fit precisely within the primary binding pocket of a recently characterized norovirus surface protein. Similarly, rotavirus spike protein VP4 contains a binding pocket with a high affinity for LNT, suggesting that LNT in milk may prevent viral binding to its target host cell moieties [136]. Thus, there is a compelling body of literature based on both structural biology and in vitro and in vivo experiments to support the direct antiviral activity of HMOs, leading some to propose that HMOs should be evaluated as a novel class of antiviral nutritionals [137].

Airflow produced during respiration, the mucocilliary action of respiratory epithelium, and peristalsis of the intestine generate constant flow across epithelial surfaces, which helps to clear bacteria, viruses, and other particles. To overcome this obstacle, many common bacterial pathogens utilize low-affinity, high-avidity interactions to increase the strength of adherence to epithelial surfaces. Terminal carbohydrate moieties, such as fucose and sialic acid, are ubiquitous on mucosal surfaces and provide ample opportunities for bacterial adhesion. HMOs serve a unique role as soluble decoys for these surface molecules, coating pathogens and preventing them from establishing stable adherence to epithelial surfaces [1]. The cumulative evidence for this phenomenon is summarized in Table 1. 2′-FL is arguably the most well-established example of this phenomenon and has been shown to inhibit the adhesion of *Campylobacter jejuni*, enteropathogenic *Escherichia coli*, *Salmonella enterica*, and *Pseudomonas aeruginosa* in multiple intestinal and epithelial cell culture model systems [138,139,140]. 3-FL appears to function in a similar but potentially complementary manner to inhibit adherence of *E. coli* and *P. aeruginosa* to epithelial cell lines [140]. These preclinical findings are supported by clinical research correlating the concentration of fucosylated HMOs in breast milk with reduced incidence of *Campylobacter* diarrhea [141] and *E. coli* infection [142]. Sialylated HMOs may act in a similar manner to inhibit strong bacterial adhesion. 3′-SL has been shown to prevent adherence of *Helicobacter pylori* to cultured gastric epithelial cells [143], and a primate trial later demonstrated that 3′-SL could assist in clearing persistent *H. pylori* infection [144].

In addition to reducing the rate of bacterial adherence through receptor decoy activity, HMOs have other direct effects that disrupt bacterial pathogenesis. Both 2′-FL and LNT exhibit extensive binding to secreted bacterial toxins produced by *E. coli* and *Vibrio cholerae*, potentially reducing their ability to inflict host damage [145]. Similarly, sialylated HMOs prevent cholera-toxin-induced diarrhea in animal models [146]. 3′-SL and 6′-SL enhance clearance of *P. aeruginosa* infection in mice, and there is evidence that the coating of *P. aeruginosa* with these HMOs may facilitate rapid endocytosis by resident tissue macrophages [147]. Additional research has shown that pooled HMOs effectively suppress the growth and formation of biofilms by *Streptococcus agalactiae* (group B *Streptococcus*, GBS) [148]. Follow-up studies clearly demonstrated that while some acetylated HMOs, such as LNT and LNnT, and complex fucosylated HMOs played an outsized role in this inhibition, the cumulative effect of pooled HMOs in both inhibition of GBS biofilm formation and direct bacterostatic activity was greater than the apparent sum of the effects of individual HMOs [149]. Thus, the binding of HMOs to bacterial pathogens represents a multi-faceted and non-specific mechanism that reduces the burden of pathogenic microbes on epithelial surfaces and promotes their clearance by innate immune cells. A rich pool of HMOs representing fucosylated and other neutral oligosaccharides as well as sialylated HMOs offers comprehensive anti-adhesive protection for developing mucosal surfaces.

#### 3.2.2. Immune Modulation

The direct action of HMOs to prevent pathogen binding to mucosal surfaces reduces the overall microbial burden. These same HMOs often act to stimulate effective cellular immunity, which greatly enhances the overall efficacy and efficiency of the immune response. Recent data examining the effects of 3′-SL and 6′-SL demonstrates the multifaceted role that HMOs play in enhancing innate immunity [147]. The presence of 3′-SL or 6′-SL stimulates robust chemokine expression and immune cell recruitment to sites of infection. Once innate immune cells arrive at the site of infection, coating of bacteria with sialylated HMOs makes these pathogens more susceptible to phagocytosis [147]. These phagocytosed bacteria are efficiently destroyed as a result of the upregulation of reactive oxygen species production stimulated by the binding of sialylated HMOs to cell surface receptors expressed by macrophages [147]. Other immune signaling pathways have also been explored. 3′-SL is also a ligand for CD33, a cell surface receptor specific to the megakaryocyte, and acts as a potent activator of their differentiation into circulating platelets [157]. Lectins are frequently proposed as potential immune cell receptors for circulating HMO [33]. LNT binds to galectin-8, a key receptor involved in defense against intracellular bacterial pathogens [158]. Both 2′-FL and 3-FL bind the dendritic cell-specific cell surface receptor DC-SIGN with a high degree of specificity, potentially modulating the behavior of these key drivers of adaptive immune cell maturation [159]. Key animal studies demonstrated that 3′-SL in milk serves as a direct stimulant for dendritic cells present in the mesenteric lymph nodes, resulting in signaling that expands T-cell populations and may contribute to the maturation of mucosal immunity [160]. Suckling rats fed supplemental 2′-FL exhibit elevated plasma IgG and IgA, suggesting that the presence of HMOs boosts the function of the developing adaptive immune system [161].

Overly responsive immune defense can itself be damaging, particularly in infants [162]. 2′-FL, 3′-SL, and 6′-SL suppress release of inflammatory cytokines associated with acute viral infection in epithelial cell culture models [131]. 3′-SL suppresses inflammatory signaling in both intestinal epithelial cells [163] and in a variety of mesenchymal cell culture models [164,165]. This may occur through the modulation of Toll-like Receptor signaling, a network of cell surface receptors that detect the presence of conserved viral and microbial components [166]. For example, He et al. demonstrated that 2′-FL suppresses CD14, a key co-factor required for the induction of inflammation following engagement of TLR4 with its ligand, lipopolysaccharide (LPS). This effectively mitigates the damaging inflammatory response that would otherwise result in bacterial pathogenesis during enterotoxigenic *E. coli* infection [167]. Both 2′-FL and 6′-SL have been shown to interact directly with TLR-4-MD2 complex to inhibit inflammatory NF-κB signaling [168]. These types of interactions may have lasting beneficial effects on the function of immune cells. In a clinical study examining > 400 healthy infants who were either exclusively breastfed, fed control formula with prebiotic galactooligosaccharides (GOS), or fed a formula containing 2′-FL and GOS, both breastfed infants and infants fed the formula containing 2′-FL exhibited lower plasma concentrations of IL-1ra, IL-1α, IL-1β, IL-6, and TNF-α compared to infants fed control formula [18]. Peripheral blood mononuclear cells (PBMCs) collected from infants who participated in this study differed in their response to ex-vivo RSV infection, with PBMCs from breastfed or 2′-FL fed infants showing reduced inflammatory cytokine expression relative to PBMCs from infants who were fed a control formula [18]. The same clinical study also showed that infants fed a formula with 2′-FL had fewer parent reports of respiratory infections [20]. This evidence suggests that not only do HMOs serve as acute modulators of immune signaling but that their presence defines and informs the complex immune environment with the potential to shape both immediate and long-term health outcomes.

#### 3.2.3. Mitigating Inflammation Associated with Preterm Birth

Perhaps the best example of the potential of HMOs as systemic immune mediators comes from studies examining the use of HMOs as therapeutic nutrients in models of necrotizing enterocolitis. Necrotizing enterocolitis (NEC) is a leading cause of morbidity and mortality in premature infants. NEC is characterized by the loss of epithelial barrier integrity and subsequent tissue inflammation and necrosis [169]. NEC affects 1% of all newborns in the United States [170], with mortality occurring in up to 30% of cases [171]. NEC risk is elevated seven-fold among premature and low-birth-weight infants [172], and the prevalence of NEC may be increasing over time [173,174]. Studies of tissue from NEC patients indicate the loss of epithelial barrier integrity and hyperacute inflammation [67,69,175]. Infants who develop NEC are more frequently colonized with certain types of bacteria [68,176,177]. This suggests a multifactorial etiology by which immature intestinal barrier function predisposes the preterm infant to intestinal injury and inflammation following postpartum microbial colonization [68,69,169].

However, the precise mechanistic basis of NEC remains elusive, and there are few treatment options available. Breastfeeding or access to human milk through a milk bank remains both the best strategy to prevent NEC in premature infants as well as a critical component of disease management in patients who develop NEC [178,179,180]. HMOs promote the growth of beneficial bacteria, suppress bacterial and viral pathogens, and have potent anti-inflammatory and immunomodulatory effects [1,33]. In an animal model of NEC, HMOs reduced intestinal barrier permeability and increased mucus production [120]. Administration of 2′-FL alone is sufficient to protect against NEC in rodents, lowering inflammatory markers and altering the composition of the intestinal microbiota [181]. Further studies demonstrated that both 2′-FL and 6′-SL reduced inflammation and apoptosis in a piglet model of NEC, often considered the gold-standard animal model of NEC [168], and that 2′-FL improved the integrity of the epithelial barrier in human intestinal tissue cultured ex vivo [182]. These experimental data are strikingly consistent with epidemiological data demonstrating that premature infants who carried the non-secretor trait had a much higher risk of morbidity and mortality due to NEC [46]. Following a similar model of investigation, early studies implicated DSLNT as an additional protective HMO in rodent models of NEC [183]. More recent epidemiological work has associated low levels of DSLNT in breastmilk with higher risk of NEC [184,185]. Thus, in the near future, supplementation of breastmilk or formula with HMOs may be a viable strategy for reducing the incidence of NEC among premature and low-birth-weight infants [186].

#### 3.2.4. Managing and Reducing the Risk of Allergy

The first two years of life represent a critical window for immune education [187]. Exposure to microoorganisms and the introduction of dietary antigens present challenges to the developing immune system. Successful navigation of these challenges results in robust adaptive immune defense as well as tolerance to dietary antigens and beneficial microbes [188]. The increasing incidence of allergic disease and asthma among children has been linked to changes in diet, environmental pollutants, and widespread hygienic practices that limit antigen exposure [70,189]. Traditional infant formulas lacking HMOs have been associated with higher risk of dietary allergy in comparison to breastfeeding, particularly cow’s milk allergy [5]. Likewise, asthma development later in childhood has also been associated with mode of feeding in infancy [72]. Given the immunomodulatory properties of HMOs, several investigations have sought to establish whether HMO fortification might offer substantive benefits for the prevention of allergic disease and asthma. Landmark work by Castillo-Courtade et al. demonstrated that dietary supplementation with 2′-FL and 6′-SL attenuated IgE-mediated anaphylactic symptoms in ovalbumin sensitized mice [190]. An increase in IL-10-expressing T regulatory cells in these mice suggested that a pro-tolerogenic immune environment formed in response to dietary HMO supplementation [190]. Further studies showed that 2′-FL and 6′-SL act on intestinal epithelial cells through distinct but complementary mechanisms to inhibit the inflammatory response that results from antigen-IgE conjugation [191].

Emerging clinical evidence may validate observations made in preclinical models suggesting that HMOs reduce the risk of allergic disease. C-section born, allergy-prone, breastfed infants whose mothers express FUT2, resulting in robust 2′-FL production in milk, are at a significantly lower risk of developing IgE-mediated eczema at two years of age [192]. This effect may apply to formula-fed infants as well. In a large-scale trial evaluating the safety and tolerability of 2′-FL fortified infant formula, Marriage et al. observed a significantly lower incidence of eczema among infants fed 2′-FL-enriched formula compared to their counterparts who received control infant formula [19]. Another recent study examined levels of 19 HMOs in 285 mothers and evaluated the risk of allergic disease in their offspring [52]. The authors observed extensive variation in HMO profiles among the study subjects but noted that the infants of mothers who expressed a rich HMO pool that was biased towards higher concentrations of neutral HMOs (2′-FL, 3-FL, LNT, etc.) relative to acidic HMOs (3′-SL, 6′-SL) had a reduced risk of asthma at 18 years of age [52]. Future studies are needed to evaluate the incidence of allergic disease in the context of infant formula fortified with similar complex mixtures of HMOs.

### 3.3. Cognitive Development

Among the more exciting and novel benefits associated with HMOs has been the discovery that HMOs may contribute significantly to cognitive development. From pre-conception through infancy and childhood, complete nutrition is among the most essential factors in ensuring healthy brain development [193]. A broad base of evidence demonstrates that breastfeeding confers substantial benefits to cognitive function in childhood even after adjustment for multiple complex confounding factors [194,195]. These benefits compound with time and likely extend into adulthood [196]. Thus, identifying the components of breastmilk that contribute to cognitive development is critical for the derivation of optimized infant formula fortification protocols.

Fucosylated proteins are present along the neural synapses of the brain, especially within the hippocampus, where they play an important role in the formation of memory and learning [197]. In vitro studies have demonstrated that supplementation with 2′-FL enhances the excitatory potential of hippocampal neurons [198]. Bienenstock et al. showed that fucosylated HMOs stimulate enteric neurons, and Goehring et al. demonstrated that 2′-FL, 3-FL, and 6′-SL in breast milk are present in plasma and/or urine [125,199]. As a result, it seems plausible that HMOs present in breastmilk or infant formula may be distributed systemically and may potentially confer direct benefits to cognitive function. Extensive follow-up studies conducted in both mice and rats showed that chronic oral administration of 2′-FL resulted in substantial improvements in spatial learning, working memory, and operant conditioning. This functional data coincided with improved long-term potentiation of hippocampal neurons and elevated expression of multiple synaptic proteins associated with memory retention [200]. These benefits extended into maturity: rats fed supplemental 2′-FL during the suckling period exhibited improved performance in spatial reasoning and object recognition tasks immediately after weaning and at one year of age [201]. Although clinical and epidemiological studies linking nutrition to developmental outcomes are inherently complex, there is recent evidence to suggest that increased exposure to 2′-FL in the early months of life is correlated with improved performance in tests of infant cognitive development [202] and motor skills [203]. Similarly, increased abundance of fucosylated HMOs in breastmilk is associated with improved language skills at 12 and 18 months of age [204].

Sialylated glycoproteins are richly distributed on cell surfaces and as a component of the extracellular matrix throughout the brain [205]. Animal models indicate that exogenous sialic acid supplementation in early life is associated with increased deposition of sialylated glycoproteins and gangliosides in the brain [206] and has positive effects on learning [207]. Breastfed infants exhibit significantly higher levels of sialic acid deposition within the frontal cortex compared to formula-fed infants, suggesting that sialylated HMOs in breastmilk are an important source of sialic acid found in brain tissue [208]. This was confirmed by an animal study showing that supplementation of formula with either 3′-SL or 6′-SL resulted in enriched sialic acid deposition within the brain tissue of suckling piglets [209]. Additional animal work revealed changes in white matter composition and connectivity in pigs fed 3′-SL [210]. A recent study found that a combination of 3′-SL and 6′-SL altered multiple brain metabolites and neurotransmitters in suckling piglets compared to control-fed littermates [211]. Together, these reports associating sialylated HMOs with changes in brain structure and signaling suggest that sialylated HMOs promote cognitive function in the developing brain. A study conducted by Tarr et al. examined the response of immature mice to social disruption stress and found that mice fed a diet supplemented with either 3′-SL or 6′-SL were highly resistant to the development of both behavioral and molecular indicators of anxiety following exposure to stressful conditions [92]. Likewise, suckling rats fed supplemental 6′-SL had improved scores on multiple cognitive tests at one year of age compared to control-fed animals or rats fed sialic acid [212]. 3′-SL promotes myelination within the hippocampus, leading to improved spatial cognition in preterm piglets [213]. Recent clinical studies have demonstrated an association between elevated levels of sialylated HMOs in breastmilk and advanced language skills at 12 and 18 months of age [204] and a specific association between increased 6′-SL concentration in breastmilk and improved cognitive and motor skills at 18 months of age [203]. Together, these studies indicate that many of the benefits seen in preclinical experiments likely apply to human infants. Thus, there is a robust body of evidence demonstrating that sialylated HMOs are associated with broad molecular and cellular changes in the developing brain and appear to play an essential role in promoting cognitive development.

There is a growing appreciation for the role of the intestinal microbiota beyond the digestive tract, including the proposal of a complex signaling relationship between gut microbes and the central nervous system known as the gut–brain axis [214,215]. Modulation of the gut–brain axis for improved learning and cognitive development outcomes is a highly promising area of investigation [214]. Given that HMOs serve as uniquely potent prebiotics, several researchers have proposed that HMOs in breastmilk confer additional indirect benefits to brain development through their effects on the composition and function of the intestinal microbiota [92]. Evidence to support this intriguing hypothesis is beginning to emerge. While a portion of dietary HMOs is clearly absorbed and distributed systemically [199], radiolabeling experiments in rodents indicated that much of the ingested HMO appears in the feces as microbial metabolites [216,217]. However, a portion of these metabolic byproducts are systemically available and potentially cross the blood–brain barrier, which may account for some of the beneficial cognitive effects of 2′-FL [216] and 3′-SL [217]. Groundbreaking work by Vázquez et al. demonstrated that while dietary 2′-FL improved hippocampal long-term potentiation and elevated scores on associated functional learning assessments, bisection of the distal portion of the vagus nerve abrogated functional benefits associated with dietary 2′-FL supplementation [218]. This study suggests that 2′-FL and/or microbial metabolites thereof act via the vagus nerve to enhance cognitive function. As our scientific understanding of the gut–brain axis continues to expand, there will be new opportunities to examine the impact of HMOs and their role in promoting beneficial microbial activity that supports brain development in infancy [219].

## 4. Summary

There now exists a large body of work defining the discrete functions of various HMOs found in human breastmilk. As detailed above, these HMOs play essential roles in promoting digestive health and the development of the microbiome, provide both direct and indirect immune protection, and support cognitive function in the developing brain. Perhaps most important, several HMOs have been proven to be safe and suitable for infants either as a supplement to breastfeeding or as a replacement when breastfeeding is not possible. There may be specific indications, such as NEC, where HMOs have an elevated importance and in which their use as supplements should be strongly considered. Recapitulating the full range of functional benefits is foremost in considering which HMOs should be part of a set of core structures utilized in the continual improvement of infant formula fortification. In addition, simple HMO structures composed of 3–6 monosaccharides are both the most common and abundant in human milk [22] and the most practical structures to produce at large scale for infant formula fortification [11,12]. An HMO fortification scheme composed of fucosylated, sialylated, and acetylated structures may deliver broad ranging benefits to formula fed infants and be practically achievable and economically viable in the near term. The core set of HMOs proposed in Figure 1, composed of 2′-FL, 3-FL, 3′-SL, 6′-SL, and LNT, would encompass a majority of the benefits described above and come closer than ever before to delivering a wide portion of the protection associated with breastfeeding. Indeed, this set of HMOs has been demonstrated to be safe and well tolerated in a recent multicenter clinical trial conducted in healthy full term infants up to four months of age [16].

**Figure 3:**
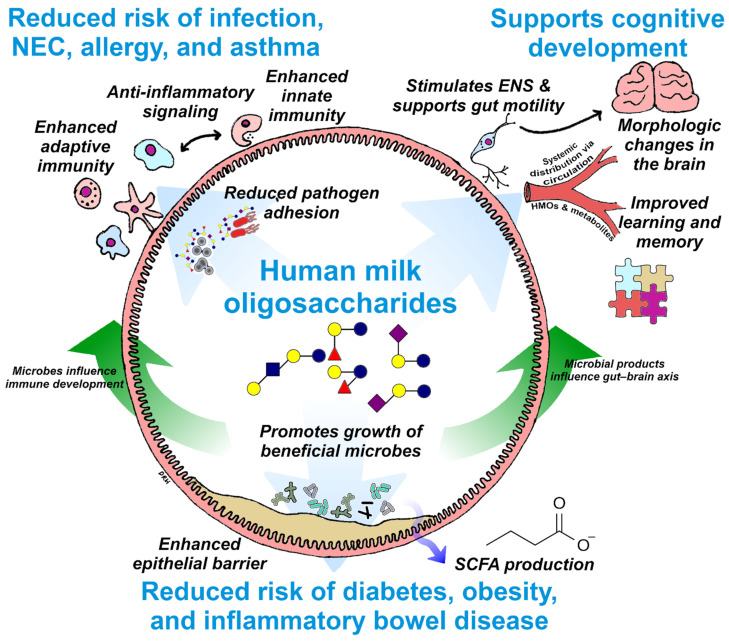
Summary of the known beneficial effects of HMOs in supporting digestive health, immunity, and cognition and an illustration of the interconnections between these benefits. Potential impacts on infant health, such as reduced risk of chronic disease and enhanced cognitive development, are highlighted in blue text. ENS, enteric nervous system; SCFA, short chain fatty acid; NEC, necrotizing enterocolitis; HMO, human milk oligosaccharides.

Innovations that recapitulate the diversity of HMO structures found naturally in breastmilk promise to yield crucial gains for digestive and immune health and cognitive development of formula fed infants (Figure 3). New studies on the role of HMOs in healthy development will continue to emerge in the years ahead. Critical questions are expected to be addressed by this research, including clarification of the application of HMO fortification in preterm infants or infants with NEC. Long-term studies may well demonstrate a role for early-life HMO fortification in the prevention of chronic diseases with immune and metabolic components, such as allergy, asthma, and type 1 diabetes. Likewise, the full benefits of early-life nutrition to cognitive development may not fully manifest for years or even decades. The continued expansion of HMO fortification and the characterization of its effects on infant health will remain perhaps the most significant development in infant nutrition for some time to come.

## Figures and Tables

**Table 1:** Summary of primary research manuscripts demonstrating that specific HMOs inhibit pathogen adhesion to host cells. Individual HMO structures exhibit distinct pathogen-binding specificity; this suggests that multiple diverse HMO structures expand the cumulative profile of anti-adhesive activity in milk. HMO, human milk oligosaccharides.

*Pathogen*	2′-FL 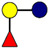	3-FL 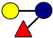	LNT 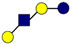	3′-SL 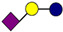	6′-SL 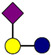
*Campylobacter jejuni*	[139,140,150]				
Enteropathogenic *E. coli*	[140,145,151]	[140]		[152]	[151]
*Vibrio cholerae* toxin			[145]	[146]	
*Salmonella enterica*	[140]	[140]			
*Helicobacter pylori*				[143,144]	[143]
Group B *Streptococcus*			[153]		
*Pseudomonas aeruginosa*				[147]	[147]
*Entamoeba histolytica*			[154]		
Respiratory Syncytial virus	[131]				
Influenza virus					[131]
Norovirus	[135,155]	[135]			
Rotavirus	[156]			[134,156]	[134,156]

## References

[1] Newburg D.S., Walker W.A. (2007). Protection of the Neonate by the Innate Immune System of Developing Gut and of Human Milk. Pediatr. Res..

[2] Frank N.M., Lynch K.F., Uusitalo U., Yang J., Lönnrot M., Virtanen S.M., Hyöty H., Norris J.M., Rewers M., Bautista K. (2019). The Relationship between Breastfeeding and Reported Respiratory and Gastrointestinal Infection Rates in Young Children. BMC Pediatr..

[3] Nishimura T., Suzue J., Kaji H. (2009). Breastfeeding Reduces the Severity of Respiratory Syncytial Virus Infection among Young Infants: A Multi-Center Prospective Study. Pediatr. Int..

[4] Schnitzer M.E., van der Laan M.J., Moodie E.E.M., Platt R.W. (2014). Effect of Breastfeeding on Gastrointestinal Infection in Infants: A Targeted Maximum Likelihood Approach for Clustered Longitudinal Data. Ann. Appl. Stat..

[5] Hays T., Wood R.A. (2005). A Systematic Review of the Role of Hydrolyzed Infant Formulas in Allergy Prevention. Arch. Pediatr. Adolesc. Med..

[6] Armstrong J., Reilly J.J. (2002). Breastfeeding and Lowering the Risk of Childhood Obesity. Lancet.

[7] Horwood L.J., Fergusson D.M. (1998). Breastfeeding and Later Cognitive and Academic Outcomes. Pediatrics.

[8] Haroon S., Das J.K., Salam R.A., Imdad A., Bhutta Z.A. (2013). Breastfeeding Promotion Interventions and Breastfeeding Practices: A Systematic Review. BMC Public Health.

[9] Section on Breastfeeding (2012). Breastfeeding and the Use of Human Milk. Pediatrics.

[10] Office of the Surgeon General (US), Centers for Disease Control and Prevention (US), Office on Women’s Health (US) (2011). Barriers to Breastfeeding in the United States.

[11] Walsh C., Lane J.A., van Sinderen D., Hickey R.M. (2020). From Lab Bench to Formulated Ingredient: Characterization, Production, and Commercialization of Human Milk Oligosaccharides. J. Funct. Foods.

[12] Zeuner B., Jers C., Mikkelsen J.D., Meyer A.S. (2014). Methods for Improving Enzymatic Trans-Glycosylation for Synthesis of Human Milk Oligosaccharide Biomimetics. J. Agric. Food Chem..

[13] Vandenplas Y., Berger B., Carnielli V.P., Ksiazyk J., Lagström H., Sanchez Luna M., Migacheva N., Mosselmans J.-M., Picaud J.-C., Possner M. (2018). Human Milk Oligosaccharides: 2′-Fucosyllactose (2′-FL) and Lacto-N-Neotetraose (LNnT) in Infant Formula. Nutrients.

[14] Nowak-Wegrzyn A., Czerkies L., Reyes K., Collins B., Heine R.G. (2019). Confirmed Hypoallergenicity of a Novel Whey-Based Extensively Hydrolyzed Infant Formula Containing Two Human Milk Oligosaccharides. Nutrients.

[15] Puccio G., Alliet P., Cajozzo C., Janssens E., Corsello G., Sprenger N., Wernimont S., Egli D., Gosoniu L., Steenhout P. (2017). Effects of Infant Formula With Human Milk Oligosaccharides on Growth and Morbidity: A Randomized Multicenter Trial. J. Pediatr. Gastroenterol. Nutr..

[16] Parschat K., Melsaether C., Jäpelt K.R., Jennewein S. (2021). Clinical Evaluation of 16-Week Supplementation with 5HMO-Mix in Healthy-Term Human Infants to Determine Tolerability, Safety, and Effect on Growth. Nutrients.

[17] Ramirez-Farias C., Baggs G.E., Marriage B.J. (2021). Growth, Tolerance, and Compliance of Infants Fed an Extensively Hydrolyzed Infant Formula with Added 2′-FL Fucosyllactose (2′-FL) Human Milk Oligosaccharide. Nutrients.

[18] Goehring K.C., Marriage B.J., Oliver J.S., Wilder J.A., Barrett E.G., Buck R.H. (2016). Similar to Those Who Are Breastfed, Infants Fed a Formula Containing 2′-Fucosyllactose Have Lower Inflammatory Cytokines in a Randomized Controlled Trial. J. Nutr..

[19] Marriage B.J., Buck R.H., Goehring K.C., Oliver J.S., Williams J.A. (2015). Infants Fed a Lower Calorie Formula with 2′FL Show Growth and 2′FL Uptake Like Breast-Fed Infants. J. Pediatr. Gastroenterol. Nutr..

[20] Reverri E.J., Devitt A.A., Kajzer J.A., Baggs G.E., Borschel M.W. (2018). Review of the Clinical Experiences of Feeding Infants Formula Containing the Human Milk Oligosaccharide 2′-Fucosyllactose. Nutrients.

[21] Storm H.M., Shepard J., Czerkies L.M., Kineman B., Cohen S.S., Reichert H., Carvalho R. (2019). 2′-Fucosyllactose Is Well Tolerated in a 100% Whey, Partially Hydrolyzed Infant Formula With Bifidobacterium Lactis: A Randomized Controlled Trial. Glob. Pediatr. Health.

[22] Thurl S., Munzert M., Boehm G., Matthews C., Stahl B. (2017). Systematic Review of the Concentrations of Oligosaccharides in Human Milk. Nutr. Rev..

[23] McGuire M.K., Meehan C.L., McGuire M.A., Williams J.E., Foster J., Sellen D.W., Kamau-Mbuthia E.W., Kamundia E.W., Mbugua S., Moore S.E. (2017). What’s Normal? Oligosaccharide Concentrations and Profiles in Milk Produced by Healthy Women Vary Geographically. Am. J. Clin. Nutr..

[24] Yu H., Yan X., Autran C.A., Li Y., Etzold S., Latasiewicz J., Robertson B.M., Li J., Bode L., Chen X. (2017). Enzymatic and Chemoenzymatic Syntheses of Disialyl Glycans and Their Necrotizing Enterocolitis Preventing Effects. J. Org. Chem..

[25] Bode L., Donovan S.M., German J.B., Lönnerdal B., Lucas A. (2019). Human Milk Oligosaccharides: Next-Generation Functions and Questions. Nestlé Nutrition Institute Workshop Series.

[26] Özcan E., Sela D.A. (2018). Inefficient Metabolism of the Human Milk Oligosaccharides Lacto-N-Tetraose and Lacto-N-Neotetraose Shifts Bifidobacterium Longum Subsp. Infantis Physiology. Front. Nutr..

[27] Eiwegger T., Stahl B., Haidl P., Schmitt J., Boehm G., Dehlink E., Urbanek R., Szépfalusi Z. (2010). Prebiotic Oligosaccharides: In Vitro Evidence for Gastrointestinal Epithelial Transfer and Immunomodulatory Properties. Pediatr. Allergy Immunol..

[28] Aldredge D.L., Geronimo M.R., Hua S., Nwosu C.C., Lebrilla C.B., Barile D. (2013). Annotation and Structural Elucidation of Bovine Milk Oligosaccharides and Determination of Novel Fucosylated Structures. Glycobiology.

[29] Fong B., Ma K., McJarrow P. (2011). Quantification of Bovine Milk Oligosaccharides Using Liquid Chromatography–Selected Reaction Monitoring–Mass Spectrometry. J. Agric. Food Chem..

[30] Gopal P.K., Gill H.S. (2000). Oligosaccharides and Glycoconjugates in Bovine Milk and Colostrum. Br. J. Nutr..

[31] Tao N., Wu S., Kim J., An H.J., Hinde K., Power M.L., Gagneux P., German J.B., Lebrilla C.B. (2011). Evolutionary Glycomics: Characterization of Milk Oligosaccharides in Primates. J. Proteome Res..

[32] Urashima T., Fukuda K., Messer M. (2012). Evolution of Milk Oligosaccharides and Lactose: A Hypothesis. Animal.

[33] Bode L. (2012). Human Milk Oligosaccharides: Every Baby Needs a Sugar Mama. Glycobiology.

[34] Bode L. (2015). The Functional Biology of Human Milk Oligosaccharides. Early Hum. Dev..

[35] German J.B., Freeman S.L., Lebrilla C.B., Mills D.A. (2008). Human Milk Oligosaccharides: Evolution, Structures and Bioselectivity as Substrates for Intestinal Bacteria. Nestle Nutr. Workshop Ser. Pediatr. Program..

[36] Ayechu-Muruzabal V., van Stigt A.H., Mank M., Willemsen L.E.M., Stahl B., Garssen J., van’t Land B. (2018). Diversity of Human Milk Oligosaccharides and Effects on Early Life Immune Development. Front. Pediatr..

[37] Chaturvedi P., Warren C.D., Altaye M., Morrow A.L., Ruiz-Palacios G., Pickering L.K., Newburg D.S. (2001). Fucosylated Human Milk Oligosaccharides Vary between Individuals and over the Course of Lactation. Glycobiology.

[38] Ma L., McJarrow P., Jan Mohamed H.J.B., Liu X., Welman A., Fong B.Y. (2018). Lactational Changes in the Human Milk Oligosaccharide Concentration in Chinese and Malaysian Mothers’ Milk. Int. Dairy J..

[39] Radzanowski G.G., Garrett P.N., Li X., Anita M. (2013). Short-Chain Milk Oligosaccharide Levels in Human Milk and Infant Plasma. FASEB J..

[40] Kumazaki T., Yoshida A. (1984). Biochemical Evidence That Secretor Gene, Se, Is a Structural Gene Encoding a Specific Fucosyltransferase. Proc. Natl. Acad. Sci. USA.

[41] Johnson P.H., Watkins W.M. (1992). Purification of the Lewis Blood-Group Gene Associated Alpha-3/4-Fucosyltransferase from Human Milk: An Enzyme Transferring Fucose Primarily to Type 1 and Lactose-Based Oligosaccharide Chains. Glycoconj. J..

[42] Ferrer-Admetlla A., Sikora M., Laayouni H., Esteve A., Roubinet F., Blancher A., Calafell F., Bertranpetit J., Casals F. (2009). A Natural History of FUT2 Polymorphism in Humans. Mol. Biol. Evol..

[43] Azad M.B., Wade K.H., Timpson N.J. (2018). FUT2 Secretor Genotype and Susceptibility to Infections and Chronic Conditions in the ALSPAC Cohort. Wellcome Open Res..

[44] Bustamante M., Standl M., Bassat Q., Vilor-Tejedor N., Medina-Gomez C., Bonilla C., Ahluwalia T.S., Bacelis J., Bradfield J.P., Tiesler C.M.T. (2016). A Genome-Wide Association Meta-Analysis of Diarrhoeal Disease in Young Children Identifies FUT2 Locus and Provides Plausible Biological Pathways. Hum. Mol. Genet..

[45] Carlsson B., Kindberg E., Buesa J., Rydell G.E., Lidón M.F., Montava R., Abu Mallouh R., Grahn A., Rodríguez-Díaz J., Bellido J. (2009). The G428A Nonsense Mutation in FUT2 Provides Strong but Not Absolute Protection against Symptomatic GII.4 Norovirus Infection. PLoS ONE.

[46] Morrow A.L., Meinzen-Derr J., Huang P., Schibler K.R., Cahill T., Keddache M., Kallapur S.G., Newburg D.S., Tabangin M., Warner B.B. (2011). Fucosyltransferase 2 Non-Secretor and Low Secretor Status Predicts Severe Outcomes in Premature Infants. J. Pediatr..

[47] Mottram L., Wiklund G., Larson G., Qadri F., Svennerholm A.-M. (2017). FUT2 Non-Secretor Status is Associated with Altered Susceptibility to Symptomatic Enterotoxigenic Escherichia Coli Infection in Bangladeshis. Sci. Rep..

[48] Thorven M., Grahn A., Hedlund K.-O., Johansson H., Wahlfrid C., Larson G., Svensson L. (2005). A Homozygous Nonsense Mutation (428G-->A) in the Human Secretor (FUT2) Gene Provides Resistance to Symptomatic Norovirus (GGII) Infections. J. Virol..

[49] Tian C., Hromatka B.S., Kiefer A.K., Eriksson N., Noble S.M., Tung J.Y., Hinds D.A. (2017). Genome-Wide Association and HLA Region Fine-Mapping Studies Identify Susceptibility Loci for Multiple Common Infections. Nat. Commun..

[50] Turpin W., Bedrani L., Espin-Garcia O., Xu W., Silverberg M.S., Smith M.I., Guttman D.S., Griffiths A., Moayyedi P., Panaccione R. (2018). FUT2 Genotype and Secretory Status Are Not Associated with Fecal Microbial Composition and Inferred Function in Healthy Subjects. Gut Microbes.

[51] Samuel T.M., Binia A., de Castro C.A., Thakkar S.K., Billeaud C., Agosti M., Al-Jashi I., Costeira M.J., Marchini G., Martínez-Costa C. (2019). Impact of Maternal Characteristics on Human Milk Oligosaccharide Composition over the First 4 Months of Lactation in a Cohort of Healthy European Mothers. Sci. Rep..

[52] Lodge C.J., Lowe A.J., Milanzi E., Bowatte G., Abramson M.J., Tsimiklis H., Axelrad C., Robertson B., Darling A.E., Svanes C. (2020). Human Milk Oligosaccharide Profiles and Allergic Disease up to 18 Years. J. Allergy Clin. Immunol..

[53] Biddulph C., Holmes M., Kuballa A., Davies P.S.W., Koorts P., Carter R.J., Maher J. (2021). Human Milk Oligosaccharide Profiles and Associations with Maternal Nutritional Factors: A Scoping Review. Nutrients.

[54] Stahl B., Thurl S., Henker J., Siegel M., Finke B., Sawatzki G. (2001). Detection of Four Human Milk Groups with Respect to Lewis-Blood-Group-Dependent Oligosaccharides by Serologic and Chromatographic Analysis. Adv. Exp. Med. Biol..

[55] Kim J., Unger S. (2010). Human Milk Banking. Paediatr. Child Health.

[56] Leaf A., Winterson R. (2009). Breast-Milk Banking: Evidence of Benefit. Paediatr. Child Health.

[57] Coppa G.V., Pierani P., Zampini L., Carloni I., Carlucci A., Gabrielli O. (1999). Oligosaccharides in Human Milk during Different Phases of Lactation. Acta Paediatr. Suppl..

[58] Thurl S., Munzert M., Henker J., Boehm G., Müller-Werner B., Jelinek J., Stahl B. (2010). Variation of Human Milk Oligosaccharides in Relation to Milk Groups and Lactational Periods. Br. J. Nutr..

[59] Austin S., De Castro C.A., Bénet T., Hou Y., Sun H., Thakkar S.K., Vinyes-Pares G., Zhang Y., Wang P. (2016). Temporal Change of the Content of 10 Oligosaccharides in the Milk of Chinese Urban Mothers. Nutrients.

[60] Kunz C., Meyer C., Collado M.C., Geiger L., García-Mantrana I., Bertua-Ríos B., Martínez-Costa C., Borsch C., Rudloff S. (2017). Influence of Gestational Age, Secretor, and Lewis Blood Group Status on the Oligosaccharide Content of Human Milk. J. Pediatr. Gastroenterol. Nutr..

[61] Sprenger N., Lee L.Y., De Castro C.A., Steenhout P., Thakkar S.K. (2017). Longitudinal Change of Selected Human Milk Oligosaccharides and Association to Infants’ Growth, an Observatory, Single Center, Longitudinal Cohort Study. PLoS ONE.

[62] Tonon K.M., Miranda A., Abrão A.C.F.V., de Morais M.B., Morais T.B. (2019). Validation and Application of a Method for the Simultaneous Absolute Quantification of 16 Neutral and Acidic Human Milk Oligosaccharides by Graphitized Carbon Liquid Chromatography-Electrospray Ionization-Mass Spectrometry. Food Chem..

[63] Urashima T., Messer M., Pontarotti P. (2017). Evolution of Milk Oligosaccharides and Their Function in Monotremes and Marsupials. Evolutionary Biology: Self/Nonself Evolution, Species and Complex Traits Evolution, Methods and Concepts.

[64] Lynch S.V., Pedersen O. (2016). The Human Intestinal Microbiome in Health and Disease. N. Eng. J. Med..

[65] Sun J., Chang E.B. (2014). Exploring Gut Microbes in Human Health and Disease: Pushing the Envelope. Genes Dis..

[66] Milani C., Duranti S., Bottacini F., Casey E., Turroni F., Mahony J., Belzer C., Delgado Palacio S., Arboleya Montes S., Mancabelli L. (2017). The First Microbial Colonizers of the Human Gut: Composition, Activities, and Health Implications of the Infant Gut Microbiota. Microbiol. Mol. Biol. Rev..

[67] Hackam D.J., Good M., Sodhi C.P. (2013). Mechanisms of Gut Barrier Failure in the Pathogenesis of Necrotizing Enterocolitis: Toll-like Receptors Throw the Switch. Semin. Pediatr. Surg..

[68] Morrow A.L., Lagomarcino A.J., Schibler K.R., Taft D.H., Yu Z., Wang B., Altaye M., Wagner M., Gevers D., Ward D.V. (2013). Early Microbial and Metabolomic Signatures Predict Later Onset of Necrotizing Enterocolitis in Preterm Infants. Microbiome.

[69] Tanner S.M., Berryhill T.F., Ellenburg J.L., Jilling T., Cleveland D.S., Lorenz R.G., Martin C.A. (2015). Pathogenesis of Necrotizing Enterocolitis: Modeling the Innate Immune Response. Am. J. Pathol..

[70] Amarasekera M., Prescott S.L., Palmer D.J. (2013). Nutrition in Early Life, Immune-Programming and Allergies: The Role of Epigenetics. Asian Pac. J. Allergy Immunol..

[71] Fujimura K.E., Lynch S.V. (2015). Microbiota in Allergy and Asthma and the Emerging Relationship with the Gut Microbiome. Cell Host Microbe.

[72] Klopp A., Vehling L., Becker A.B., Subbarao P., Mandhane P.J., Turvey S.E., Lefebvre D.L., Sears M.R., Daley D., Silverman F. (2017). Modes of Infant Feeding and the Risk of Childhood Asthma: A Prospective Birth Cohort Study. J. Pediatr..

[73] Cox L.M., Blaser M.J. (2015). Antibiotics in Early Life and Obesity. Nat. Rev. Endocrinol..

[74] Schulfer A.F., Schluter J., Zhang Y., Brown Q., Pathmasiri W., McRitchie S., Sumner S., Li H., Xavier J.B., Blaser M.J. (2019). The Impact of Early-Life Sub-Therapeutic Antibiotic Treatment (STAT) on Excessive Weight Is Robust despite Transfer of Intestinal Microbes. ISME J..

[75] Kronman M.P., Zaoutis T.E., Haynes K., Feng R., Coffin S.E. (2012). Antibiotic Exposure and IBD Development among Children: A Population-Based Cohort Study. Pediatrics.

[76] Troelsen F.S., Jick S. (2020). Antibiotic Use in Childhood and Adolescence and Risk of Inflammatory Bowel Disease: A Case-Control Study in the UK Clinical Practice Research Datalink. Inflamm. Bowel. Dis..

[77] David L.A., Maurice C.F., Carmody R.N., Gootenberg D.B., Button J.E., Wolfe B.E., Ling A.V., Devlin A.S., Varma Y., Fischbach M.A. (2014). Diet Rapidly and Reproducibly Alters the Human Gut Microbiome. Nature.

[78] Wu G.D., Chen J., Hoffmann C., Bittinger K., Chen Y.-Y., Keilbaugh S.A., Bewtra M., Knights D., Walters W.A., Knight R. (2011). Linking Long-Term Dietary Patterns with Gut Microbial Enterotypes. Science.

[79] Baumann-Dudenhoeffer A.M., D’Souza A.W., Tarr P.I., Warner B.B., Dantas G. (2018). Infant Diet and Maternal Gestational Weight Gain Predict Early Metabolic Maturation of Gut Microbiomes. Nat. Med..

[80] Timmerman H.M., Rutten N.B.M.M., Boekhorst J., Saulnier D.M., Kortman G.A.M., Contractor N., Kullen M., Floris E., Harmsen H.J.M., Vlieger A.M. (2017). Intestinal Colonisation Patterns in Breastfed and Formula-Fed Infants during the First 12 Weeks of Life Reveal Sequential Microbiota Signatures. Sci. Rep..

[81] Gibson G.R., Hutkins R., Sanders M.E., Prescott S.L., Reimer R.A., Salminen S.J., Scott K., Stanton C., Swanson K.S., Cani P.D. (2017). Expert Consensus Document: The International Scientific Association for Probiotics and Prebiotics (ISAPP) Consensus Statement on the Definition and Scope of Prebiotics. Nat. Rev. Gastroenterol. Hepatol..

[82] Asakuma S., Hatakeyama E., Urashima T., Yoshida E., Katayama T., Yamamoto K., Kumagai H., Ashida H., Hirose J., Kitaoka M. (2011). Physiology of Consumption of Human Milk Oligosaccharides by Infant Gut-Associated Bifidobacteria. J. Biol. Chem..

[83] Garrido D., Ruiz-Moyano S., Lemay D.G., Sela D.A., German J.B., Mills D.A. (2015). Comparative Transcriptomics Reveals Key Differences in the Response to Milk Oligosaccharides of Infant Gut-Associated Bifidobacteria. Sci. Rep..

[84] Thongaram T., Hoeflinger J.L., Chow J., Miller M.J. (2017). Human Milk Oligosaccharide Consumption by Probiotic and Human-Associated Bifidobacteria and Lactobacilli. J. Dairy Sci..

[85] Ruiz-Moyano S., Totten S.M., Garrido D.A., Smilowitz J.T., German J.B., Lebrilla C.B., Mills D.A. (2013). Variation in Consumption of Human Milk Oligosaccharides by Infant Gut-Associated Strains of Bifidobacterium Breve. Appl. Environ. Microbiol..

[86] Marcobal A., Barboza M., Froehlich J.W., Block D.E., German J.B., Lebrilla C.B., Mills D.A. (2010). Consumption of Human Milk Oligosaccharides by Gut-Related Microbes. J. Agric. Food Chem..

[87] Yu Z.-T., Chen C., Kling D.E., Liu B., McCoy J.M., Merighi M., Heidtman M., Newburg D.S. (2013). The Principal Fucosylated Oligosaccharides of Human Milk Exhibit Prebiotic Properties on Cultured Infant Microbiota. Glycobiology.

[88] Yu Z.-T., Chen C., Newburg D.S. (2013). Utilization of Major Fucosylated and Sialylated Human Milk Oligosaccharides by Isolated Human Gut Microbes. Glycobiology.

[89] Bauer M.A., Kainz K., Carmona-Gutierrez D., Madeo F. (2019). Microbial Wars: Competition in Ecological Niches and within the Microbiome. Microb. Cell.

[90] Korpela K., Salonen A., Hickman B., Kunz C., Sprenger N., Kukkonen K., Savilahti E., Kuitunen M., de Vos W.M. (2018). Fucosylated Oligosaccharides in Mother’s Milk Alleviate the Effects of Caesarean Birth on Infant Gut Microbiota. Sci. Rep..

[91] Berger B., Porta N., Foata F., Grathwohl D., Delley M., Moine D., Charpagne A., Siegwald L., Descombes P., Alliet P. (2020). Linking Human Milk Oligosaccharides, Infant Fecal Community Types, and Later Risk To Require Antibiotics. mBio.

[92] Tarr A.J., Galley J.D., Fisher S.E., Chichlowski M., Berg B.M., Bailey M.T. (2015). The Prebiotics 3′Sialyllactose and 6′Sialyllactose Diminish Stressor-Induced Anxiety-like Behavior and Colonic Microbiota Alterations: Evidence for Effects on the Gut-Brain Axis. Brain Behav. Immun..

[93] Marcobal A., Sonnenburg J.L. (2012). Human Milk Oligosaccharide Consumption by Intestinal Microbiota. Clin. Microbiol. Infect..

[94] D’Souza G., Shitut S., Preussger D., Yousif G., Waschina S., Kost C. (2018). Ecology and Evolution of Metabolic Cross-Feeding Interactions in Bacteria. Nat. Prod. Rep..

[95] Gotoh A., Katoh T., Sakanaka M., Ling Y., Yamada C., Asakuma S., Urashima T., Tomabechi Y., Katayama-Ikegami A., Kurihara S. (2018). Sharing of Human Milk Oligosaccharides Degradants within Bifidobacterial Communities in Faecal Cultures Supplemented with Bifidobacterium Bifidum. Sci. Rep..

[96] Lawson M.A.E., O’Neill I.J., Kujawska M., Gowrinadh Javvadi S., Wijeyesekera A., Flegg Z., Chalklen L., Hall L.J. (2020). Breast Milk-Derived Human Milk Oligosaccharides Promote Bifidobacterium Interactions within a Single Ecosystem. ISME J..

[97] Koh A., De Vadder F., Kovatcheva-Datchary P., Bäckhed F. (2016). From Dietary Fiber to Host Physiology: Short-Chain Fatty Acids as Key Bacterial Metabolites. Cell.

[98] Liu H., Wang J., He T., Becker S., Zhang G., Li D., Ma X. (2018). Butyrate: A Double-Edged Sword for Health?. Adv. Nutr..

[99] Chia L.W., Mank M., Blijenberg B., Bongers R.S., Aalvink S., van Limpt K., Wopereis H., Tims S., Stahl B., Belzer C. (2018). Cross-Feeding between Bifidobacterium Infantis and Anaerostipes Caccae on Lactose and Human Milk Oligosaccharides. bioRxiv.

[100] Van den Abbeele P., Duysburgh C., Vazquez E., Chow J., Buck R., Marzorati M. (2019). 2′-Fucosyllactose Alters the Composition and Activity of Gut Microbiota from Formula-Fed Infants Receiving Complementary Feeding in a Validated Intestinal Model. J. Funct. Foods.

[101] Vester Boler B.M., Rossoni Serao M.C., Faber T.A., Bauer L.L., Chow J., Murphy M.R., Fahey G.C. (2013). In Vitro Fermentation Characteristics of Select Nondigestible Oligosaccharides by Infant Fecal Inocula. J. Agric. Food Chem..

[102] Yang B., Chen Y., Stanton C., Ross R.P., Lee Y.-K., Zhao J., Zhang H., Chen W. (2019). Bifidobacterium and Lactobacillus Composition at Species Level and Gut Microbiota Diversity in Infants before 6 Weeks. Int. J. Mol. Sci..

[103] Zúñiga M., Monedero V., Yebra M.J. (2018). Utilization of Host-Derived Glycans by Intestinal Lactobacillus and Bifidobacterium Species. Front. Microbiol..

[104] Salli K., Hirvonen J., Siitonen J., Ahonen I., Anglenius H., Maukonen J. (2021). Selective Utilization of the Human Milk Oligosaccharides 2′-Fucosyllactose, 3-Fucosyllactose, and Difucosyllactose by Various Probiotic and Pathogenic Bacteria. J. Agric. Food Chem..

[105] Kaiko G.E., Ryu S.H., Koues O.I., Collins P.L., Solnica-Krezel L., Pearce E.J., Pearce E.L., Oltz E.M., Stappenbeck T.S. (2016). The Colonic Crypt Protects Stem Cells from Microbiota-Derived Metabolites. Cell.

[106] Sun Y., O’Riordan M.X.D. (2013). Regulation of Bacterial Pathogenesis by Intestinal Short-Chain Fatty Acids. Adv. Appl. Microbiol..

[107] Fukuda S., Toh H., Hase K., Oshima K., Nakanishi Y., Yoshimura K., Tobe T., Clarke J.M., Topping D.L., Suzuki T. (2011). Bifidobacteria Can Protect from Enteropathogenic Infection through Production of Acetate. Nature.

[108] Bondue P., Crèvecoeur S., Brose F., Daube G., Seghaye M.-C., Griffiths M.W., LaPointe G., Delcenserie V. (2016). Cell-Free Spent Media Obtained from Bifidobacterium Bifidum and Bifidobacterium Crudilactis Grown in Media Supplemented with 3′-Sialyllactose Modulate Virulence Gene Expression in Escherichia Coli O157:H7 and Salmonella Typhimurium. Front. Microbiol..

[109] Kiu R., Treveil A., Harnisch L.C., Caim S., Leclaire C., van Sinderen D., Korcsmaros T., Hall L.J. (2020). Bifidobacterium Breve UCC2003 Induces a Distinct Global Transcriptomic Program in Neonatal Murine Intestinal Epithelial Cells. iScience.

[110] Bergmann K.R., Liu S.X.L., Tian R., Kushnir A., Turner J.R., Li H.-L., Chou P.M., Weber C.R., De Plaen I.G. (2013). Bifidobacteria Stabilize Claudins at Tight Junctions and Prevent Intestinal Barrier Dysfunction in Mouse Necrotizing Enterocolitis. Am. J. Pathol..

[111] Ewaschuk J.B., Diaz H., Meddings L., Diederichs B., Dmytrash A., Backer J., Looijer-van Langen M., Madsen K.L. (2008). Secreted Bioactive Factors from Bifidobacterium Infantis Enhance Epithelial Cell Barrier Function. Am. J. Physiol. Gastrointest Liver Physiol..

[112] Guo S., Gillingham T., Guo Y., Meng D., Zhu W., Walker W.A., Ganguli K. (2017). Secretions of Bifidobacterium Infantis and Lactobacillus Acidophilus Protect Intestinal Epithelial Barrier Function. J. Pediatr. Gastroenterol. Nutr..

[113] Šuligoj T., Vigsnæs L.K., den Abbeele P.V., Apostolou A., Karalis K., Savva G.M., McConnell B., Juge N. (2020). Effects of Human Milk Oligosaccharides on the Adult Gut Microbiota and Barrier Function. Nutrients.

[114] Meng D., Sommella E., Salviati E., Campiglia P., Ganguli K., Djebali K., Zhu W., Walker W.A. (2020). Indole-3-Lactic Acid, a Metabolite of Tryptophan, Secreted by Bifidobacterium Longum Subspecies Infantis Is Anti-Inflammatory in the Immature Intestine. Pediatr. Res..

[115] Chichlowski M., De Lartigue G., German J.B., Raybould H.E., Mills D.A. (2012). Bifidobacteria Isolated from Infants and Cultured on Human Milk Oligosaccharides Affect Intestinal Epithelial Function. J. Pediatr. Gastroenterol. Nutr..

[116] Xiao L., Van’t Land B., Engen P.A., Naqib A., Green S.J., Nato A., Leusink-Muis T., Garssen J., Keshavarzian A., Stahl B. (2018). Human Milk Oligosaccharides Protect against the Development of Autoimmune Diabetes in NOD-Mice. Sci. Rep..

[117] Grabinger T., Glaus Garzon J.F., Hausmann M., Geirnaert A., Lacroix C., Hennet T. (2019). Alleviation of Intestinal Inflammation by Oral Supplementation With 2-Fucosyllactose in Mice. Front. Microbiol..

[118] Lee S., Goodson M., Vang W., Kalanetra K., Barile D., Raybould H. (2020). 2′-Fucosyllactose Supplementation Improves Gut-Brain Signaling and Diet-Induced Obese Phenotype and Changes the Gut Microbiota in High Fat-Fed Mice. Nutrients.

[119] Cheng L., Kong C., Walvoort M.T.C., Faas M.M., de Vos P. (2020). Human Milk Oligosaccharides Differently Modulate Goblet Cells Under Homeostatic, Proinflammatory Conditions and ER Stress. Mol. Nutr. Food Res..

[120] Wu R.Y., Li B., Koike Y., Määttänen P., Miyake H., Cadete M., Johnson-Henry K.C., Botts S.R., Lee C., Abrahamsson T.R. (2019). Human Milk Oligosaccharides Increase Mucin Expression in Experimental Necrotizing Enterocolitis. Mol. Nutr. Food Res..

[121] Kong C., Elderman M., Cheng L., de Haan B.J., Nauta A., de Vos P. (2019). Modulation of Intestinal Epithelial Glycocalyx Development by Human Milk Oligosaccharides and Non-Digestible Carbohydrates. Mol. Nutr. Food Res..

[122] Holscher H.D., Davis S.R., Tappenden K.A. (2014). Human Milk Oligosaccharides Influence Maturation of Human Intestinal Caco-2Bbe and HT-29 Cell Lines. J. Nutr..

[123] Natividad J.M., Rytz A., Keddani S., Bergonzelli G., Garcia-Rodenas C.L. (2020). Blends of Human Milk Oligosaccharides Confer Intestinal Epithelial Barrier Protection in Vitro. Nutrients.

[124] Herath M., Hosie S., Bornstein J.C., Franks A.E., Hill-Yardin E.L. (2020). The Role of the Gastrointestinal Mucus System in Intestinal Homeostasis: Implications for Neurological Disorders. Front. Cell. Infect. Microbiol..

[125] Bienenstock J., Buck R.H., Linke H., Forsythe P., Stanisz A.M., Kunze W.A. (2013). Fucosylated but Not Sialylated Milk Oligosaccharides Diminish Colon Motor Contractions. PLoS ONE.

[126] Farhin S., Wong A., Delungahawatta T., Amin J.Y., Bienenstock J., Buck R., Kunze W.A. (2019). Restraint Stress Induced Gut Dysmotility Is Diminished by a Milk Oligosaccharide (2′-Fucosyllactose) in Vitro. PLoS ONE.

[127] Burrell C.J., Howard C.R., Murphy F.A. (2017). Epidemiology of Viral Infections. Fenner White Med. Virol..

[128] Kieninger E., Fuchs O., Latzin P., Frey U., Regamey N. (2013). Rhinovirus Infections in Infancy and Early Childhood. Eur. Respir. J..

[129] Strodtbeck F. (1995). Viral Infections of the Newborn. J. Obstet. Gynecol. Neonatal. Nurs..

[130] Tregoning J.S., Schwarze J. (2010). Respiratory Viral Infections in Infants: Causes, Clinical Symptoms, Virology, and Immunology. Clin. Microbiol. Rev..

[131] Duska-McEwen G., Senft A.P., Ruetschilling T.L., Barrett E.G., Buck R.H. (2014). Human Milk Oligosaccharides Enhance Innate Immunity to Respiratory Syncytial Virus and Influenza in Vitro. FNS.

[132] Simoes E.A. (1999). Respiratory Syncytial Virus Infection. Lancet.

[133] Morrow A.L., Ruiz-Palacios G.M., Jiang X., Newburg D.S. (2005). Human-Milk Glycans That Inhibit Pathogen Binding Protect Breast-Feeding Infants against Infectious Diarrhea. J. Nutr..

[134] Hester S.N., Chen X., Li M., Monaco M.H., Comstock S.S., Kuhlenschmidt T.B., Kuhlenschmidt M.S., Donovan S.M. (2013). Human Milk Oligosaccharides Inhibit Rotavirus Infectivity in Vitro and in Acutely Infected Piglets. Br. J. Nutr..

[135] Weichert S., Koromyslova A., Singh B.K., Hansman S., Jennewein S., Schroten H., Hansman G.S. (2016). Structural Basis for Norovirus Inhibition by Human Milk Oligosaccharides. J. Virol..

[136] Hu L., Ramani S., Czako R., Sankaran B., Yu Y., Smith D.F., Cummings R.D., Estes M.K., Venkataram Prasad B.V. (2015). Structural Basis of Glycan Specificity in Neonate-Specific Bovine-Human Reassortant Rotavirus. Nat. Commun..

[137] Morozov V., Hansman G., Hanisch F.-G., Schroten H., Kunz C. (2018). Human Milk Oligosaccharides as Promising Antivirals. Mol. Nutr. Food Res..

[138] Newburg D.S., Pickering L.K., McCluer R.H., Cleary T.G. (1990). Fucosylated Oligosaccharides of Human Milk Protect Suckling Mice from Heat-Stabile Enterotoxin of Escherichia Coli. J. Infect. Dis..

[139] Ruiz-Palacios G.M., Cervantes L.E., Ramos P., Chavez-Munguia B., Newburg D.S. (2003). Campylobacter Jejuni Binds Intestinal H(O) Antigen (Fuc Alpha 1, 2Gal Beta 1, 4GlcNAc), and Fucosyloligosaccharides of Human Milk Inhibit Its Binding and Infection. J. Biol. Chem..

[140] Weichert S., Jennewein S., Hüfner E., Weiss C., Borkowski J., Putze J., Schroten H. (2013). Bioengineered 2′-Fucosyllactose and 3-Fucosyllactose Inhibit the Adhesion of Pseudomonas Aeruginosa and Enteric Pathogens to Human Intestinal and Respiratory Cell Lines. Nutr. Res..

[141] Morrow A.L., Ruiz-Palacios G.M., Altaye M., Jiang X., Guerrero M.L., Meinzen-Derr J.K., Farkas T., Chaturvedi P., Pickering L.K., Newburg D.S. (2004). Human Milk Oligosaccharides Are Associated with Protection against Diarrhea in Breast-Fed Infants. J. Pediatr..

[142] Newburg D.S., Ruiz-Palacios G.M., Altaye M., Chaturvedi P., Guerrero M.L., Meinzen-Derr J.K., Morrow A.L. (2004). Human Milk Alphal,2-Linked Fucosylated Oligosaccharides Decrease Risk of Diarrhea Due to Stable Toxin of E. Coli in Breastfed Infants. Adv. Exp. Med. Biol..

[143] Simon P.M., Goode P.L., Mobasseri A., Zopf D. (1997). Inhibition of Helicobacter Pylori Binding to Gastrointestinal Epithelial Cells by Sialic Acid-Containing Oligosaccharides. Infect. Immun..

[144] Mysore J.V., Wigginton T., Simon P.M., Zopf D., Heman-Ackah L.M., Dubois A. (1999). Treatment of Helicobacter Pylori Infection in Rhesus Monkeys Using a Novel Antiadhesion Compound. Gastroenterology.

[145] El-Hawiet A., Kitova E.N., Klassen J.S. (2015). Recognition of Human Milk Oligosaccharides by Bacterial Exotoxins. Glycobiology.

[146] Idota T., Kawakami H., Murakami Y., Sugawara M. (1995). Inhibition of Cholera Toxin by Human Milk Fractions and Sialyllactose. Biosci. Biotechnol. Biochem..

[147] Kim J., Kim Y.-J., Kim J.W. (2019). Bacterial Clearance Is Enhanced by A2,3- and A2,6-Sialyllactose via Receptor-Mediated Endocytosis and Phagocytosis. Infect. Immun..

[148] Ackerman D.L., Doster R.S., Weitkamp J.-H., Aronoff D.M., Gaddy J.A., Townsend S.D. (2017). Human Milk Oligosaccharides Exhibit Antimicrobial and Antibiofilm Properties against Group B *Streptococcus*. ACS Infect. Dis..

[149] Craft K.M., Thomas H.C., Townsend S.D. (2018). Interrogation of Human Milk Oligosaccharide Fucosylation Patterns for Antimicrobial and Antibiofilm Trends in Group B *Streptococcus*. ACS Infect. Dis..

[150] Yu Z.-T., Nanthakumar N.N., Newburg D.S. (2016). The Human Milk Oligosaccharide 2′-Fucosyllactose Quenches Campylobacter Jejuni-Induced Inflammation in Human Epithelial Cells HEp-2 and HT-29 and in Mouse Intestinal Mucosa. J. Nutr..

[151] Facinelli B., Marini E., Magi G., Zampini L., Santoro L., Catassi C., Monachesi C., Gabrielli O., Coppa G.V. (2019). Breast Milk Oligosaccharides: Effects of 2′-Fucosyllactose and 6′-Sialyllactose on the Adhesion of Escherichia Coli and Salmonella Fyris to Caco-2 Cells. J. Matern. Fetal. Neonatal. Med..

[152] Angeloni S., Ridet J.L., Kusy N., Gao H., Crevoisier F., Guinchard S., Kochhar S., Sigrist H., Sprenger N. (2005). Glycoprofiling with Micro-Arrays of Glycoconjugates and Lectins. Glycobiology.

[153] Lin A.E., Autran C.A., Szyszka A., Escajadillo T., Huang M., Godula K., Prudden A.R., Boons G.-J., Lewis A.L., Doran K.S. (2017). Human Milk Oligosaccharides Inhibit Growth of Group B *Streptococcus*. J. Biol. Chem..

[154] Jantscher-Krenn E., Lauwaet T., Bliss L.A., Reed S.L., Gillin F.D., Bode L. (2012). Human Milk Oligosaccharides Reduce Entamoeba Histolytica Attachment and Cytotoxicity in Vitro. Br. J. Nutr..

[155] Koromyslova A., Tripathi S., Morozov V., Schroten H., Hansman G.S. (2017). Human Norovirus Inhibition by a Human Milk Oligosaccharide. Virology.

[156] Laucirica D.R., Triantis V., Schoemaker R., Estes M.K., Ramani S. (2017). Milk Oligosaccharides Inhibit Human Rotavirus Infectivity in MA104 Cells. J. Nutr..

[157] Ha S.-H., Kwak C.-H., Park J.-Y., Abekura F., Lee Y.-C., Kim J.-S., Chung T.-W., Kim C.-H. (2020). 3′-Sialyllactose Targets Cell Surface Protein, SIGLEC-3, and Induces Megakaryocyte Differentiation and Apoptosis by Lipid Raft-Dependent Endocytosis. Glycoconj. J..

[158] Bohari M.H., Yu X., Zick Y., Blanchard H. (2016). Structure-Based Rationale for Differential Recognition of Lacto- and Neolacto- Series Glycosphingolipids by the N-Terminal Domain of Human Galectin-8. Sci. Rep..

[159] Noll A.J., Yu Y., Lasanajak Y., Duska-McEwen G., Buck R.H., Smith D.F., Cummings R.D. (2016). Human DC-SIGN Binds Specific Human Milk Glycans. Biochem. J..

[160] Kurakevich E., Hennet T., Hausmann M., Rogler G., Borsig L. (2013). Milk Oligosaccharide Sialyl(A2,3)Lactose Activates Intestinal CD11c+ Cells through TLR4. Proc. Natl. Acad. Sci. USA.

[161] Azagra-Boronat I., Massot-Cladera M., Mayneris-Perxachs J., Knipping K., van’t Land B., Tims S., Stahl B., Garssen J., Franch À., Castell M. (2019). Immunomodulatory and Prebiotic Effects of 2′-Fucosyllactose in Suckling Rats. Front. Immunol..

[162] Frost B.L., Jilling T., Caplan M.S. (2008). The Importance of Pro-Inflammatory Signaling in Neonatal Necrotizing Enterocolitis. Semin. Perinatol..

[163] Zenhom M., Hyder A., de Vrese M., Heller K.J., Roeder T., Schrezenmeir J. (2011). Prebiotic Oligosaccharides Reduce Proinflammatory Cytokines in Intestinal Caco-2 Cells via Activation of PPARγ and Peptidoglycan Recognition Protein 3. J. Nutr..

[164] Jeon J., Kang L.-J., Lee K.M., Cho C., Song E.K., Kim W., Park T.J., Yang S. (2018). 3′-Sialyllactose Protects against Osteoarthritic Development by Facilitating Cartilage Homeostasis. J. Cell Mol. Med..

[165] Kang L.-J., Kwon E.-S., Lee K.M., Cho C., Lee J.-I., Ryu Y.B., Youm T.H., Jeon J., Cho M.R., Jeong S.-Y. (2018). 3′-Sialyllactose as an Inhibitor of P65 Phosphorylation Ameliorates the Progression of Experimental Rheumatoid Arthritis. Br. J. Pharmacol..

[166] He Y., Lawlor N.T., Newburg D.S. (2016). Human Milk Components Modulate Toll-Like Receptor–Mediated Inflammation12. Adv. Nutr..

[167] He Y., Liu S., Kling D.E., Leone S., Lawlor N.T., Huang Y., Feinberg S.B., Hill D.R., Newburg D.S. (2016). The Human Milk Oligosaccharide 2′-Fucosyllactose Modulates CD14 Expression in Human Enterocytes, Thereby Attenuating LPS-Induced Inflammation. Gut.

[168] Sodhi C.P., Wipf P., Yamaguchi Y., Fulton W.B., Kovler M., Niño D.F., Zhou Q., Banfield E., Werts A.D., Ladd M.R. (2020). The Human Milk Oligosaccharides 2′-Fucosyllactose and 6′-Sialyllactose Protect against the Development of Necrotizing Enterocolitis by Inhibiting Toll-like Receptor 4 Signaling. Pediatr. Res..

[169] Neu J., Walker W.A. (2011). Necrotizing Enterocolitis. N. Eng. J. Med..

[170] Holman R.C., Stoll B.J., Curns A.T., Yorita K.L., Steiner C.A., Schonberger L.B. (2006). Necrotising Enterocolitis Hospitalisations among Neonates in the United States. Paediatr. Perinat Epidemiol..

[171] Lin P.W., Stoll B.J. (2006). Necrotising Enterocolitis. Lancet.

[172] Boccia D., Stolfi I., Lana S., Moro M.L. (2001). Nosocomial Necrotising Enterocolitis Outbreaks: Epidemiology and Control Measures. Eur. J. Pediatr..

[173] Gephart S.M., McGrath J.M., Effken J.A., Halpern M.D. (2012). Necrotizing Enterocolitis Risk: State of the Science. Adv. Neonatal. Care.

[174] Schullinger J.N., Mollitt D.L., Vinocur C.D., Santulli T.V., Driscoll J.M. (1981). Neonatal Necrotizing Enterocolitis. Survival, Management, and Complications: A 25-Year Study. Am. J. Dis. Child..

[175] Nanthakumar N., Meng D., Goldstein A.M., Zhu W., Lu L., Uauy R., Llanos A., Claud E.C., Walker W.A. (2011). The Mechanism of Excessive Intestinal Inflammation in Necrotizing Enterocolitis: An Immature Innate Immune Response. PLoS ONE.

[176] Torrazza R.M., Ukhanova M., Wang X., Sharma R., Hudak M.L., Neu J., Mai V. (2013). Intestinal Microbial Ecology and Environmental Factors Affecting Necrotizing Enterocolitis. PLoS ONE.

[177] Ward D.V., Scholz M., Zolfo M., Taft D.H., Schibler K.R., Tett A., Segata N., Morrow A.L. (2016). Metagenomic Sequencing with Strain-Level Resolution Implicates Uropathogenic E. Coli in Necrotizing Enterocolitis and Mortality in Preterm Infants. Cell Rep..

[178] Cañizo Vázquez D., Salas García S., Izquierdo Renau M., Iglesias-Platas I. (2019). Availability of Donor Milk for Very Preterm Infants Decreased the Risk of Necrotizing Enterocolitis without Adversely Impacting Growth or Rates of Breastfeeding. Nutrients.

[179] Colaizy T.T., Bartick M.C., Jegier B.J., Green B.D., Reinhold A.G., Schaefer A.J., Bogen D.L., Schwarz E.B., Stuebe A.M., Jobe A.H. (2016). Impact of Optimized Breastfeeding on the Costs of Necrotizing Enterocolitis in Extremely Low Birthweight Infants. J. Pediatr..

[180] Kantorowska A., Wei J.C., Cohen R.S., Lawrence R.A., Gould J.B., Lee H.C. (2016). Impact of Donor Milk Availability on Breast Milk Use and Necrotizing Enterocolitis Rates. Pediatrics.

[181] Good M., Sodhi C.P., Yamaguchi Y., Jia H., Lu P., Fulton W.B., Martin L.Y., Prindle T., Nino D.F., Zhou Q. (2016). The Human Milk Oligosaccharide 2′-Fucosyllactose Attenuates the Severity of Experimental Necrotising Enterocolitis by Enhancing Mesenteric Perfusion in the Neonatal Intestine. Br. J. Nutr..

[182] Werts A.D., Fulton W.B., Ladd M.R., Saad-Eldin A., Chen Y.X., Kovler M.L., Jia H., Banfield E.C., Buck R.H., Goehring K. (2019). A Novel Role for Necroptosis in the Pathogenesis of Necrotizing Enterocolitis. Cell Mol. Gastroenterol. Hepatol..

[183] Jantscher-Krenn E., Zherebtsov M., Nissan C., Goth K., Guner Y.S., Naidu N., Choudhury B., Grishin A.V., Ford H.R., Bode L. (2012). The Human Milk Oligosaccharide Disialyllacto-N-Tetraose Prevents Necrotising Enterocolitis in Neonatal Rats. Gut.

[184] Masi A.C., Embleton N.D., Lamb C.A., Young G., Granger C.L., Najera J., Smith D.P., Hoffman K.L., Petrosino J.F., Bode L. (2020). Human Milk Oligosaccharide DSLNT and Gut Microbiome in Preterm Infants Predicts Necrotising Enterocolitis. Gut.

[185] Hassinger D., Clausen D.M., Nitka S., Herdt A., Griffin I. (2020). Analysis of Disialyllacto-N-Tetraose (DSLNT) Content in Milk From Mothers of Preterm Infants. J. Hum. Lact..

[186] Bering S.B. (2018). Human Milk Oligosaccharides to Prevent Gut Dysfunction and Necrotizing Enterocolitis in Preterm Neonates. Nutrients.

[187] Lloyd C.M., Saglani S. (2019). Opening the Window of Immune Opportunity: Treating Childhood Asthma. Trends Immunol..

[188] Gensollen T., Blumberg R.S. (2017). Correlation between Early Life Regulation of Immune System by Microbiota and Allergy Development. J. Allergy Clin. Immunol..

[189] Renz H., Holt P.G., Inouye M., Logan A.C., Prescott S.L., Sly P.D. (2017). An Exposome Perspective: Early-Life Events and Immune Development in a Changing World. J. Allergy Clin. Immunol..

[190] Castillo-Courtade L., Han S., Lee S., Mian F.M., Buck R., Forsythe P. (2015). Attenuation of Food Allergy Symptoms Following Treatment with Human Milk Oligosaccharides in a Mouse Model. Allergy.

[191] Zehra S., Khambati I., Vierhout M., Mian M.F., Buck R., Forsythe P. (2018). Human Milk Oligosaccharides Attenuate Antigen-Antibody Complex Induced Chemokine Release from Human Intestinal Epithelial Cell Lines. J. Food Sci..

[192] Sprenger N., Odenwald H., Kukkonen A.K., Kuitunen M., Savilahti E., Kunz C. (2017). FUT2-Dependent Breast Milk Oligosaccharides and Allergy at 2 and 5 Years of Age in Infants with High Hereditary Allergy Risk. Eur. J. Nutr..

[193] Prado E.L., Dewey K.G. (2014). Nutrition and Brain Development in Early Life. Nutr. Rev..

[194] Anderson J.W., Johnstone B.M., Remley D.T. (1999). Breast-Feeding and Cognitive Development: A Meta-Analysis. Am. J. Clin. Nutr..

[195] Schack-Nielsen L., Michaelsen K.F. (2007). Advances in Our Understanding of the Biology of Human Milk and Its Effects on the Offspring. J. Nutr..

[196] Mortensen E.L., Michaelsen K.F., Sanders S.A., Reinisch J.M. (2002). The Association between Duration of Breastfeeding and Adult Intelligence. JAMA.

[197] Murrey H.E., Gama C.I., Kalovidouris S.A., Luo W.-I., Driggers E.M., Porton B., Hsieh-Wilson L.C. (2006). Protein Fucosylation Regulates Synapsin Ia/Ib Expression and Neuronal Morphology in Primary Hippocampal Neurons. Proc. Natl. Acad. Sci. USA.

[198] Matthies H., Staak S., Krug M. (1996). Fucose and Fucosyllactose Enhance In-Vitro Hippocampal Long-Term Potentiation. Brain Res..

[199] Goehring K.C., Kennedy A.D., Prieto P.A., Buck R.H. (2014). Direct Evidence for the Presence of Human Milk Oligosaccharides in the Circulation of Breastfed Infants. PLoS ONE.

[200] Vázquez E., Barranco A., Ramírez M., Gruart A., Delgado-García J.M., Martínez-Lara E., Blanco S., Martín M.J., Castanys E., Buck R. (2015). Effects of a Human Milk Oligosaccharide, 2′-Fucosyllactose, on Hippocampal Long-Term Potentiation and Learning Capabilities in Rodents. J. Nutr. Biochem..

[201] Oliveros E., Ramirez M., Vazquez E., Barranco A., Gruart A., Delgado-Garcia J.M., Buck R., Rueda R., Martin M.J. (2016). Oral Supplementation of 2′-Fucosyllactose during Lactation Improves Memory and Learning in Rats. J. Nutr. Biochem..

[202] Berger P.K., Plows J.F., Jones R.B., Alderete T.L., Yonemitsu C., Poulsen M., Ryoo J.H., Peterson B.S., Bode L., Goran M.I. (2020). Human Milk Oligosaccharide 2′-Fucosyllactose Links Feedings at 1 Month to Cognitive Development at 24 Months in Infants of Normal and Overweight Mothers. PLoS ONE.

[203] Oliveros E., Martín M.J., Torres-Espínola F.J., Segura-Moreno M.T., Ramírez M., Santos A., Buck R., Rueda R., Escudero M., Catena A. (2021). Human Milk Levels of 2’-Fucosyllactose and 6´-Sialyllactose Are Positively Associated with Infant Neurodevelopment and Are Not Impacted by Maternal BMI or Diabetic Status. Nutrients.

[204] Jorgensen J.M., Young R., Ashorn P., Ashorn U., Chaima D., Davis J.C.C., Goonatilleke E., Kumwenda C., Lebrilla C.B., Maleta K. (2020). Associations of Human Milk Oligosaccharides and Bioactive Proteins with Infant Growth and Development among Malawian Mother-Infant Dyads. Am. J. Clin. Nutr..

[205] Schnaar R.L., Gerardy-Schahn R., Hildebrandt H. (2014). Sialic Acids in the Brain: Gangliosides and Polysialic Acid in Nervous System Development, Stability, Disease, and Regeneration. Physiol. Rev..

[206] Carlson S.E., House S.G. (1986). Oral and Intraperitoneal Administration of N-Acetylneuraminic Acid: Effect on Rat Cerebral and Cerebellar N-Acetylneuraminic Acid. J. Nutr..

[207] Morgan B.L., Winick M. (1980). Effects of Administration of N-Acetylneuraminic Acid (NANA) on Brain NANA Content and Behavior. J. Nutr..

[208] Wang B., McVeagh P., Petocz P., Brand-Miller J. (2003). Brain Ganglioside and Glycoprotein Sialic Acid in Breastfed Compared with Formula-Fed Infants. Am. J. Clin. Nutr..

[209] Jacobi S.K., Yatsunenko T., Li D., Dasgupta S., Yu R.K., Berg B.M., Chichlowski M., Odle J. (2016). Dietary Isomers of Sialyllactose Increase Ganglioside Sialic Acid Concentrations in the Corpus Callosum and Cerebellum and Modulate the Colonic Microbiota of Formula-Fed Piglets. J. Nutr..

[210] Mudd A.T., Fleming S.A., Labhart B., Chichlowski M., Berg B.M., Donovan S.M., Dilger R.N. (2017). Dietary Sialyllactose Influences Sialic Acid Concentrations in the Prefrontal Cortex and Magnetic Resonance Imaging Measures in Corpus Callosum of Young Pigs. Nutrients.

[211] Wang H.X., Chen Y., Haque Z., de Veer M., Egan G., Wang B. (2019). Sialylated Milk Oligosaccharides Alter Neurotransmitters and Brain Metabolites in Piglets: An In Vivo Magnetic Resonance Spectroscopic (MRS) Study. Nutr. Neurosci..

[212] Oliveros E., Vázquez E., Barranco A., Ramírez M., Gruart A., Delgado-García J.M., Buck R., Rueda R., Martín M.J. (2018). Sialic Acid and Sialylated Oligosaccharide Supplementation during Lactation Improves Learning and Memory in Rats. Nutrients.

[213] Obelitz-Ryom K., Bering S.B., Overgaard S.H., Eskildsen S.F., Ringgaard S., Olesen J.L., Skovgaard K., Pankratova S., Wang B., Brunse A. (2019). Bovine Milk Oligosaccharides with Sialyllactose Improves Cognition in Preterm Pigs. Nutrients.

[214] Brett B.E., de Weerth C. (2019). The Microbiota–Gut–Brain Axis: A Promising Avenue to Foster Healthy Developmental Outcomes. Dev. Psychobiol..

[215] Grenham S., Clarke G., Cryan J.F., Dinan T.G. (2011). Brain–Gut–Microbe Communication in Health and Disease. Front. Physiol..

[216] Kuntz S., Kunz C., Borsch C., Vazquez E., Buck R., Reutzel M., Eckert G.P., Rudloff S. (2019). Metabolic Fate and Distribution of 2´-Fucosyllactose: Direct Influence on Gut Microbial Activity but Not on Brain. Mol. Nutr. Food Res..

[217] Galuska C.E., Rudloff S., Kuntz S., Borsch C., Reutzel M., Eckert G., Galuska S.P., Kunz C. (2020). Metabolic Fate and Organ Distribution of 13C-3′-Sialyllactose and 13C-N-Acetylneuraminic Acid in Wild-Type Mice—No Evidence for Direct Incorporation into the Brain. J. Funct. Foods.

[218] Vazquez E., Barranco A., Ramirez M., Gruart A., Delgado-Garcia J.M., Jimenez M.L., Buck R., Rueda R. (2016). Dietary 2′-Fucosyllactose Enhances Operant Conditioning and Long-Term Potentiation via Gut-Brain Communication through the Vagus Nerve in Rodents. PLoS ONE.

[219] Al-Khafaji A.H., Jepsen S.D., Christensen K.R., Vigsnæs L.K. (2020). The Potential of Human Milk Oligosaccharides to Impact the Microbiota-Gut-Brain Axis through Modulation of the Gut Microbiota. J. Funct. Foods.

